# Profilin-1 deficiency leads to SMAD3 upregulation and impaired 3D outgrowth of breast cancer cells

**DOI:** 10.1038/s41416-018-0284-6

**Published:** 2018-10-15

**Authors:** Souvik Chakraborty, Chang Jiang, David Gau, Michael Oddo, Zhijie Ding, Laura Vollmer, Marion Joy, William Schiemann, Donna Beer Stolz, Andreas Vogt, Sujoy Ghosh, Partha Roy

**Affiliations:** 1 Bioengineering, University of Pittsburgh, Pittsburgh, USA; 2 Drug Discovery Institute, University of Pittsburgh, Pittsburgh, USA; 3 Case Western Reserve University, Cleveland, USA; 4 Cell Biology, University of Pittsburgh, Pittsburgh, USA; 5 Center for Computational Biology, Duke-NUS Medical School, Singapore, Singapore; 6 Pathology, University of Pittsburgh, Pittsburgh, USA; 7 Present Address: Harvard Medical School, Boston, MA USA; 8 Present Address: Janssen Scientific Affairs, New Jersey Raritan, USA; 9 Present Address: NSABP, Pittsburgh, PA USA

## Abstract

**Background:**

Adhesion-mediated activation of FAK/ERK signalling pathway, enabled by the formation of filopodial protrusions (FLP), has been shown to be an important event for triggering of dormancy-to-proliferation switch and metastatic outgrowth of breast cancer cells (BCC). We studied the role of actin-binding protein profilin1 (Pfn1) in these processes.

**Methods:**

Quantitative immunohistochemistry (IHC) of BC tissue microarray (TMA) and survival analyses of curated transcriptome datasets of BC patients were performed to examine Pfn1’s association with certain clinicopathological features. FLP formation and single cell outgrowth of BCC were assessed using a 3D matrigel culture that accurately predicts dormant vs metastatic outgrowth phenotypes of BCC in certain microenvironment. Gene expression studies were performed to identify potential biological pathways that are perturbed under Pfn1-depleted condition.

**Results:**

Lower Pfn1 expression is correlated with lower nuclear grade of breast tumours and longer relapse-free survival of BC patients. Pfn1 depletion leads to defects in FLP and outgrowth of BCC but without impairing either FAK or ERK activation. Guided by transcriptome analyses, we further showed that Pfn1 depletion is associated with prominent SMAD3 upregulation. Although knockdown and overexpression experiments revealed that SMAD3 has an inhibitory effect on the outgrowth of breast cancer cells, SMAD3 knockdown alone was not sufficient to enhance the outgrowth potential of Pfn1-depleted BCC suggesting that other proliferation-regulatory pathways in conjunction with SMAD3 upregulation may underlie the outgrowth-deficient phenotype of BCC cells upon depletion of Pfn1.

**Conclusion:**

Overall, these data suggest that Pfn1 may be a novel biomarker for BC recurrence and a possible target to reduce metastatic outgrowth of BCC.

## Introduction

Tumour metastasis is a multistep process that requires cancer cells to escape from the primary tumour, survive in the circulation, invade the target organs and finally reinitiate secondary tumour outgrowth at the metastatic sites. Oncogenic transformation causes alterations of the actin cytoskeleton through deregulating expression and/or activity of a wide range of actin-regulatory proteins. These changes are often correlated with acquisition of a motile phenotype of cancer cells. Recent studies have also underscored the importance of actin cytoskeleton in governing tumour initiation and metastatic outgrowth of cancer cells. For example, metastatic outgrowth of extravasated breast cancer (BC) cells (BCC) is promoted by signalling triggered by the extracellular matrix (ECM) component of the local microenvironment, and this involves changes in actin cytoskeletal architecture. Specifically, ECM-induced activation of integrin receptors potentiates actin stress-fibre assembly through a FAK (focal adhesion kinase)-Src-ERK (extracellular signalling regulated kinase) signalling axis. Preventing actin stress-fibre assembly or blocking integrin signalling dramatically inhibits FAK-Src-ERK signalling and subsequent metastatic outgrowth of disseminated BCC cells.^1–5^ Adhesion-mediated triggering of proliferation switch of BCC is enabled by the formation of F-actin-rich filopodial-like protrusions (FLPs). Disseminated BCC that fail to initiate FLPs become incompetent in metastatic outgrowth and therefore remain dormant. Furthermore, FLPs are also abundant in the sub-population of BCC that are endowed with stem-cell like features and have higher tumour-initiating potential (whether at the primary or metastatic sites) than the non-stem-like pool of BCC. Therefore, FLP abundance has been regarded a general deterministic indicator for tumourigenic ability of BCC cells. Formation of FLPs requires the actions of Rho-GTPases (Rif, Cdc42—these are responsible for activation of actin-nucleating factors) and actin nucleation and/or elongating factors such as Arp 2/3 complex, formin family protein mDia2 (mammalian diaphanous 2) and Ena/VASP (enabled/vasodilator activated phosphoprotein) proteins. Loss of function (LOF) of these actin cytoskeletal regulators reduces the incidence of primary tumours from experimentally implanted BCC as well as causes impairment in metastatic outgrowth of extravasated BCC.^6,7^ These data further suggest the general importance of actin assembly factors or their upstream regulators in outgrowth and tumour-initiating abilities (whether at the primary or the metastatic sites) of BCC.

Profilin (Pfn) family of actin-binding proteins serve as a common denominator in various cellular pathways of actin assembly including those driven by formin and Ena/VASP proteins (these proteins are capable of binding to Pfn and recruiting polymerisation-competent actin monomers via Pfn). There are two main cellular isoforms of Pfn: Pfn1 (the major isoform) and Pfn2 (with the exception of neuronal cells this minor isoform of Pfn is generally expressed at a much lower level than Pfn1).^8^ Although these two Pfn isoforms are structurally similar and generally bind to the similar types of ligands, they differ in their affinities for various ligands. Pfn1 expression is partially downregulated in the primary tumour in human BC, more prominently in those associated with distant metastasis.^9,10^ In contrast to the pro-migratory role of Pfn in most physiological contexts, there is evidence that downregulation of Pfn isoforms can induce hypermigratory phenotype in certain BCC lines.^10–13^ At least in MDA-MB-231 (MDA-231: a widely used triple-negative BC (TNBC: ER-, PR-, HER-) cell line) xenograft model, we showed in vivo evidence of increased proficiency of BCC to escape from experimentally established primary tumours upon Pfn1 depletion. Collectively, these studies implied that downregulation of Pfn1 can promote the early steps of BC metastasis (similarly, another group demonstrated increased dissemination of lung cancer cells upon LOF of Pfn1^14^). Contrasting these pro-metastatic features associated with LOF of Pfn, we showed that depletion of Pfn1 can severely impair the development of pulmonary metastases from extravasated MDA-231 cells suggesting there may be Pfn1-dependency for metastatic colonisation of TNBC cells.^10^ However, whether (a) it is the inability to survive or reduced metastatic outgrowth in the lung parenchyma that causes the colonisation defect of BCC upon Pfn1 depletion and (b) there is any potential association between Pfn1 expression and growth-related clinical features in human BC, remain unclear.

In this study, we demonstrate that lower Pfn1 expression correlates with lower nuclear grade (NG: an indicator of growth-aggressiveness of tumour cells) of BC cells and longer relapse-free survival (RFS) of BC patients. Using a three-dimensional (3D) basement membrane matrix (BME or matrigel) culture (also known as MoT (*matrigel-on-top*) culture: it closely approximates the compliant mechanical microenvironment of lung and mammary gland) that accurately predicts the metastatic outgrowth competency of BCC in the lungs,^6,7,15^ we show that Pfn1 deficiency causes defects in FLP abundance (a feature of outgrowth-competent cells) and reduces single cell outgrowth ability of TNBC cells. Our studies further suggest that Pfn1 depletion induces outgrowth defect in TNBC cells by affecting an ECM-stiffness sensitive signalling pathway that is important for BCC outgrowth other than the FAK/ERK signalling.

## Materials and methods

### Cell culture, 3D outgrowth, and FLP assay

Generation and culture of sublines of MDA-231 cells stably expressing control- and Pfn1-shRNAs have been described previously.^10,16^ MDA-157 cell line was cultured in RPMI media supplemented with antibiotics and 10% FBS. Three-dimensional (3D) outgrowth of BCC was assessed using MoT assay in either 384- or 96-well format as previously described.^6,7,15^ Essentially, cells were seeded at different densities on a layer of 100% growth factor-reduced matrigel (Cultrex, Trevigen) and then overlaid with culture medium containing 2% matrigel. In experiments involving collagen-I, matrigel was mixed with collagen-I (BD Biosciences) with final concentrations of matrigel and collagen-I equal to 7.9 mg/mL and 2 mg/mL, respectively. After 7–10 days of culture, cells were fixed overnight with 4% paraformaldehyde and 5% sucrose in PBS, permeabilised for 5 min with 4% paraformaldehyde, 5% sucrose, and 0.25% Triton X-100 in PBS before staining for 25 min with a mixture of 4% paraformaldehyde, 5% sucrose, and 10 μg/mL Hoechst 33342 (Invitrogen) in PBS followed by washing for 10 min with PBS twice. Cells were imaged on an ImageXpress Ultra confocal high content reader using a ×4 objective. A total of twenty 50 µm thick planes was acquired and collapsed using a maximum projection algorithm. Objects were enumerated using the MetaXpress multiwavelength cell scoring algorithm. For FLP assay, cells were cultured in 8 chamber glass slide system (Lab-TEK®II, Thermo Fisher Scientific) coated with 50 μL growth factor-reduced matrigel (without or with collagen-I) for 3 days. Following fixation and permeabilisation as described above, cells were incubated overnight with either Alexa Fluor 488- or rhodamine-phalloidin (Thermo Fisher Scientific, 1:250 dilution), and washed with PBS for 15 min for three times. The slides were then mounted overnight with gelvatol mounting medium in dark. The images were taken using Nikon A1 Spectral Confocal Microscope.

#### RNAi and viral infection

The sequences of single target and smart-pool siRNAs targeting Pfn1 isoforms (source: GE Dharmacon) and smart-pool of Pfn2 (source: Santa Cruz) were reported in our previous studies.^13,17^ Doxycycline (Dox)-inducible TRIPZ Pfn1 Lentiviral shRNA vector (antisense targeting sequence: CACGTAAAAACTTGACCGG; this vector also contains a turbo-RFP reporter) was purchased from Dharmacon. Adenovirus encoding EGFP (Ad-GFP) and SMAD3 (Ad-SMAD3) were obtained from University of Pittsburgh Viral core and Vector Biolabs (Malvern, PA), respectively. For SMAD3 knockdown, two different sets of siRNAs with no targeting sequence overlap were used: siRNA#1 ON-TARGETplus Human Smad3 siRNA – SMARTpool (Sequence 1: CAACAGGAAUGCAGCAGUG, Sequence 2: GAGUUCGCCUUCAAUAUGA, Sequence 3: GGACGCAGGUUCUCCAAAC, Sequence 4: UUAGAGACAUCAAGUAUGG; Thermo Fisher Scientific) and siRNA #2 Human Smad3 Stealth siRNA (Sequence: GATGCAACCTGAAGATCTTCAACAA; Thermo Fisher Scientific). All siRNA transfection was performed using transfection reagent 2 from Dharmacon. The cells were transfected with the siRNAs for 24 h before performing outgrowth assays. Adenoviral infection was performed overnight at 500 MOI before performing outgrowth assays. The efficiency of transduction was assessed by % GFP-positive cells 48–72 h after infection. Lentivirus was produced by the Magee-Women’s Hospital of University of Pittsburgh lentiviral core facility. Lentiviral infection of MDA-231 was performed at MOI of 500 in the presence of 8 μg/ml of Polybrene. Cells with the strongest RFP signal after dox induction (2 μg/ml) were selected and the best Pfn1 knockdown clone was selected for further experiments. For outgrowth experiments, cells were treated initially with dox (2 μg/ml) for 2 days before seeding on matrigel and dox was replenished every third day.

#### Collagen-1 immunostaining

Cells cultured on coverslips were fixed with 4% paraformaldehyde for 15 min at room temperature. The cells were permeabilised using 0.25% Triton X-100 in PBS, blocked with 10% goat serum in PBS, and incubated with anti-Collagen-I alpha1 antibody (Novus, 1:100 dilution) in Ab-buffer (10% goat serum/0.25% Tween 20/1X PBS) for 1 h. After primary antibody incubation, cells were washed three times with 1X PBS and incubated with Alexa 488-conjugated secondary antibody for 1 h. Cells were then washed three times with PBS before mounting on glass slides with mounting media containing DAPI for fluorescence imaging using an Olympus IX71 microscope. Fluorescence images were background subtracted, and the fluorescence intensity of collagen-I immunoreactivity was analysed on a cell-by-cell basis for computing the average fluorescence intensity based on the analyses of several randomly selected fields containing 150–200 cells pooled from two independent experiments.

### Protein extraction, immunoblotting

Total cell lysate from 2D monolayer culture was prepared by extracting cells with modified RIPA buffer (50 mM Tris-HCl, pH 7.5, 150 mM NaCl, 1% NP-40, 0.25% sodium deoxycholate, 0.1% SDS, 2 mM EDTA) supplemented with 50 mM NaF, 1 mM sodium pervanadate, and protease inhibitors. Total cell lysate from 3D culture was prepared by incubating cells at 4 °C in the modified RIPA buffer supplemented with 0.5% SDS and protease/phosphatase inhibitor cocktail (Pierce). The extracts were clarified by centrifugation at 13,000 rpm for 30 min and the supernatant was used for immunoblotting. Sources of different antibodies were: anti-Pfn1 (Abcam), anti-phospho-FAK (Y397) (Invitrogen), anti-Smad3 (Biorad), anti-ERK1/2, anti-phospho-ERK1/2 and anti-phospho-Smad3 (S423/S425) (Cell Signalling), anti-Pfn2 and anti-FAK (Santa Cruz), and anti-GAPDH and anti-Tubulin (Sigma-Aldrich) Immunoblotting concentrations for different antibodies were: 1:3000 for anti-Pfn1, anti-GAPDH and anti-tubulin; 1:500 for anti-Pfn2, anti-ERK1/2, anti-pERK1/2 and anti-FAK; and 1:1000 for anti-Smad3, anti-pSmad3 and anti-pFAK.

### Tissue microarray (TMA) analyses

We used the YTMA49–12 breast cancer TMA (source: Yale University Department of Pathology) constructed from archived invasive breast cancer patient samples from 1961 to 1983. This TMA represented ER (oestrogen receptor)-positive and ER-negative tumours approximately at a 1:1 ratio and has been used in many previous studies for cancer biomarker identification and validation.^18,19^ Fluorescence staining of TMA with anti-Pfn1, anti-pan-cytokeratin and DAPI, and quantification of Pfn1 immunostaining in cytokeratin-positive areas (representing tumour cells) were performed accordingly to a previous protocol.^20^

### Microarray data/pathway enrichment analyses

Gene expression signals from each hybridised microarray (Affymetrix platform) were derived by the MAS5 algorithm. Fold-change estimates (Pfn1 KD vs control) were calculated from each of the two independent Pfn KD experiments (transcriptomes of two different Pfn1 shRNA clones were analysed) and converted to log_2_ ratios. A total of 13,008 probes showing consistent direction of fold-changes in all experiments were retained for further analysis. Pathway enrichment analysis was conducted via 2 different approaches. First, gene-set enrichment analysis (GSEA^21^) was conducted on a list of 13,008 probes, ranked by the magnitude of their median fold-change across the 3 studies. Specifically, we used the ‘pre-ranked GSEA’ option with enrichment statistic set to ‘classic’, and queried the canonical pathway (C2:CP) and oncogenic signature databases (C6:oncogenic signatures), available from the Molecular Signature Database, MSigDb.^22^ False discovery rate (FDR) were controlled by the *q*-value and pathways with FDR ≤10% were considered to be significantly enriched. Significant pathways were further clustered based on shared gene members through the EnrichmentMap application in Cytoscape.^23^ In addition to GSEA, we employed the Ingenuity Pathway Analysis, IPA (Qiagen) tool to identify potential upstream regulators that influence gene expression upon Pfn1 knockdown. We pre-selected 349 genes with an absolute fold-change ≥1.5-fold as input for IPA. Overlap of regulator target genes with the experimental input gene list was assessed by the Fisher’s exact test; whereas, the consistency of the overlapping gene expression profiles with activation or inhibition of the regulator (predicted from prior data in the Ingenuity Knowledge Base) was quantified by the *z*-score method, as described (http://pages.ingenuity.com/rs/ingenuity/images/0812%20upstream_regulator_analysis_whitepaper.pdf). Regulators with an overlap *p*-value ≤0.01 and absolute *z*-score ≥2 were considered significantly affected by Pfn1 KD treatment.

### Survival analysis on human cancer transcriptomes

The effects of Pfn gene expression on survival outcomes in cancer patients was conducted via informatic analysis of transcriptome datasets for breast cancer patient tumour samples available from kmplot.com.^24^ Curated genomic and clinical data for each of these cancers were obtained from Gene Expression Omnibus (GEO), European Genome-Phenome Archive (EGA) and The Cancer Gene Atlas (TCGA). Overall survival(OS) or relapse-free survival(RFS) was evaluated on all available patients using the Kaplan–Meier approach, after dividing the patient populations based on the median gene expression across all samples tested. Differences in survival between two groups were compared using log-rank tests, with statistical significance ascribed at *P* < 0.01. The relative rates of time to death in the two groups (above and below median gene expression) were expressed via hazard ratios.

### General statistical analyses

Statistical differences were assessed by Student’s *T*-test or one-way ANOVA, and a *p*-value of <0.05 was considered significant. Data in bar graphs represent mean ± standard deviation(SD) values.

## Results

### Lower Pfn1 expression is associated with lower nuclear grade tumour and longer RFS in BC

**Fig. 1:**
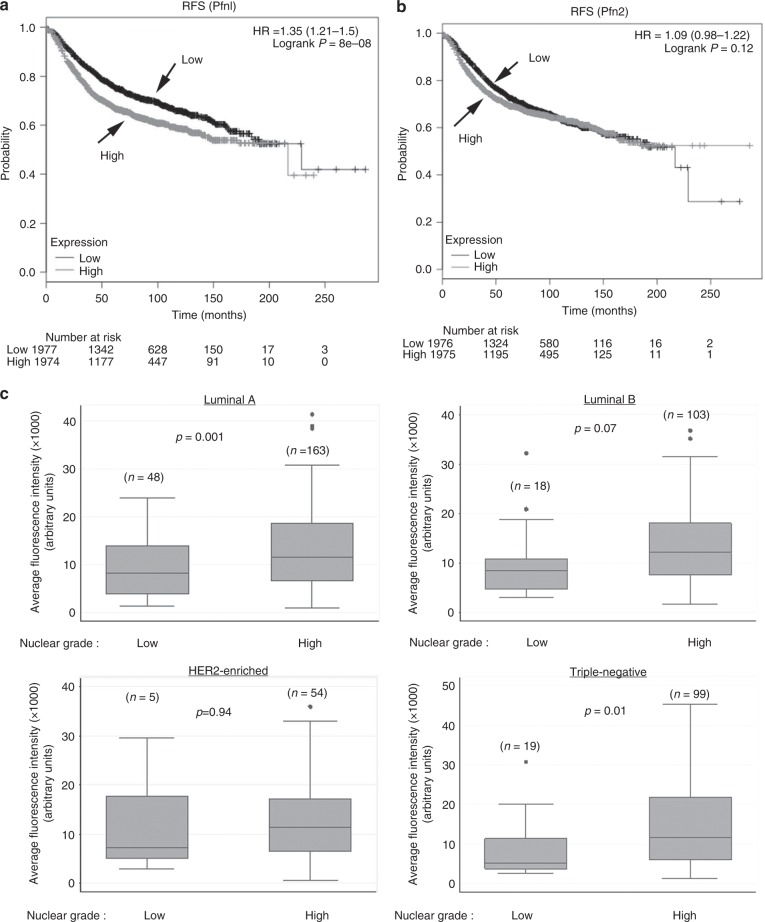
Association of Pfn1 expression with nuclear grade of tumour and RFS of BC patients. **a,b** Kaplan–Meier survival plots showing the effect of Pfn1 **(a)** and Pfn2 **(b)** gene expression (above or below median levels of expression) on RFS based on analyses of transcriptome datasets for BC tumours (3951 samples) using kmplot.com. **c** Box-whisker plots comparing relative Pfn1 expression (based on immunofluorescence analyses of Pfn1 expression in YTMA49-12 TMA) between low (*N* = 1) and high nuclear (*N* = 2 or 3) grade breast tumours for different molecular subtypes of BC (“n” indicates the number of samples in each category). In the plots, the middle line, the upper and lower hinges of the box represent the median, 75th and 25th percentile of data and the whiskers represent the maximum and minimum values

We first asked whether there is any association between Pfn1 expression and growth-related clinical features in human BC. Since BC recurrence involves either metastatic or locoregional outgrowth of therapy-resistant tumour cells, we interrogated the effects of Pfn1 and Pfn2 transcript levels on the RFS and the OS of BC patients (3951 samples) using pre-assembled transcriptome datasets available from kmplot.com. Univariate Kaplan–Meier and log-rank analyses demonstrated that although there was no significant association between the gene expression of any of the two Pfn isoforms and the OS of BC patients (supplementary Fig S1), higher Pfn1 (but not Pfn2) gene expression level (>median expression) was significantly associated with an increased risk of recurrence of BC (HR = 1.35 (1.21–1.5), logrank *P* = 8E−08) (Fig. 1a, b). Nuclear grade (NG) of BC is a good predictor for growth aggressiveness of tumour cells and high NG tumours have a higher propensity to relapse. Therefore, we next analysed Pfn1 expression at the protein level by quantitative IHC in a BC TMA consisting of a large panel (>500 samples) of primary tumours isolated from invasive BC patients and compared the relative Pfn1 expression between low nuclear grade (1) vs high nuclear grade (2 and 3) tumours (Fig. 1c). For both luminal A (ER and/or PR+, HER2−) and triple-negative (ER−, PR− and HER2−) BC, the average Pfn1 immunoreactivity was significantly lower in low NG tumours than higher nuclear grade tumours. The difference was also almost close to significance (*p* = 0.07) for luminal B (ER and/or PR+; HER2+) BC. The only exception was HER2-enriched BC (ER−, PR−, HER2+) where no significant difference in Pfn1 expression was noted between the low and the high NG tumours. Whether this deviation is due to any biological effect of HER2 signalling on Pfn1 expression or lower sample number in luminal B and HER2-enriched BC compared to the other two subtypes is not clear. Overall, these clinical correlation data support the notion of Pfn1 being an important determinant for growth aggressiveness of BCC, and suggest better patient outcome corresponding to lower Pfn1 gene expression at least in terms of BC recurrence.

### Loss-of-Pfn1 inhibits FLP and retards single-cell outgrowth of TNBC cells but without impairing the overall FAK/ERK activation

**Fig. 2:**
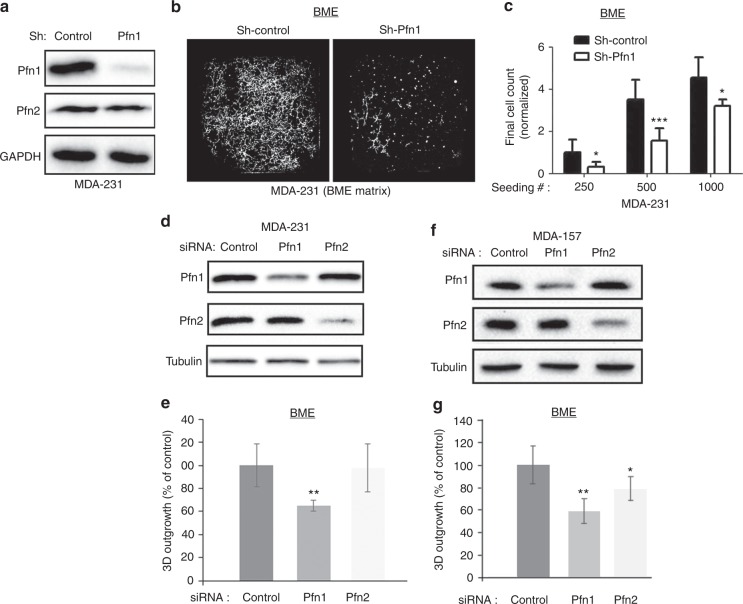
Effect of Pfn1 depletion on single cell outgrowth of BCC in 3D BME matrix. **a** Pfn1 and Pfn2 immunoblot analyses of total cell extracts of MDA-231 cells stably expressing either control or Pfn1-shRNA (GAPDH blot serves as a loading control). **b** Representative images of outgrowth of control and Pfn1-shRNA MDA-231 cells on 3D BME matrix from an initial seeding of 500 cells/well in a 384-well plate. **c** A bar graph summarizing the final number of cells (counted on day 10) in control vs Pfn1 shRNA groups for different seeding densities. All data are normalised to the final number of cells in control group for initial seeding density equal to 250 cells/well based on cell-count analyses of 3 technical replicates/condition from two independent experiments. **d-g**; **d**, **f** Immunoblot analyses of Pfn1 and Pfn2 from total cell extracts of MDA-231 (**d**) and MDA-157 (**f**) cells following transient transfection with the indicated siRNAs (tubulin blot serves as a loading control). The bar graphs in **e** and **g** summarise the outgrowth of Pfn1 and Pfn2 KD MDA-231 and MDA-157 cells, respectively, on 3D BME matrix 7 days after seeding relative to the respective control siRNA transfected cell lines (data summarised from two independent experiments with three technical replicates per experiment) *p < 0.05; **p < 0.01; ***p < 0.001). Values are presented as mean ± SD

To assess the impact of LOF of Pfn1 on the outgrowth competency of BCC, we first analysed the single cell outgrowth of sparsely seeded MDA-231 TNBC cells stably expressing either Pfn1 shRNA or a non-targeting control shRNA, as a function of seeding density in BME-MoT assay. We used MDA-231 as a model system as this cell line exhibited post-extravasation lung colonisation defect upon depletion of Pfn1 in our previous study.^10^ Immunoblot in Fig. 2a demonstrates successful knockdown (KD) of Pfn1 expression in stable Pfn1-shRNA expressing subline of MDA-231 cells (note that Pfn2 expression was unaffected when Pfn1 was stably silenced). Although stable KD of Pfn1 did not lead to proliferation defect of MDA-231 cells in 2D monolayer culture (supplementary Fig S2), it led to a significant impairment in the outgrowth ability of these cells in BME-MoT assay (Fig. 2b, c). Outgrowth kinetics revealed that the rate of outgrowth of Pfn1 shRNA cells was much slower than control cells for all seeding densities; however, the differences in their growth rates were reduced with increasing cell-seeding density as judged by the relative slopes of the growth curves (supplementary Fig S3). These findings were also reproducible in a transient transfection setting when Pfn1 expression was knocked down using a set of pooled siRNAs selective for Pfn1, and targeting sequences of which were different from the one targeted by the aforementioned Pfn1 shRNA (Fig. 2d, e). In these experiments, transient silencing of Pfn1 but not Pfn2 reduced single cell outgrowth of MDA-231 cells (note that transient KD of Pfn1 also did not affect Pfn2 expression and vice-versa). In addition, we evaluated the outgrowth of an MDA-231 subline that stably expressed GFP-Pfn1 following transfection with either a smart pool (SP) Pfn1 siRNA (knocks down both endogenous Pfn1 and exogenous GFP-Pfn1) or a single-target (ST) Pfn1 siRNA (this siRNA knocks down only endogenous Pfn1 but does not affect the expression of GFP-Pfn1 as GFP-Pfn1 was rendered siRNA-resistant through silent mutation) transfection. A statistically significant 30% higher outgrowth readout of GFP-Pfn1 expressers in ST-Pfn1 compared to SP-Pfn1 transfection settings suggests that retention of exogenous Pfn1 can augment the outgrowth ability of Pfn1-depleted MDA-231 cells (supplementary Fig S4). Finally, to extend our finding of reduced 3D outgrowth of BCC upon Pfn1 depletion to other TNBC cells, we performed similar BME-MoT experiments with MDA-MB-157 (MDA-157), another metastatic TNBC cell line. We found that MDA-157 outgrowth was reduced by knockdown of either Pfn1 or Pfn2, although outgrowth inhibition was more prominent upon depletion of Pfn1 than of Pfn2 (Fig. 2f, g). Collectively, these finding demonstrate that Pfn1 is an important determinant of outgrowth proficiency of TNBC cells.

**Fig. 3:**
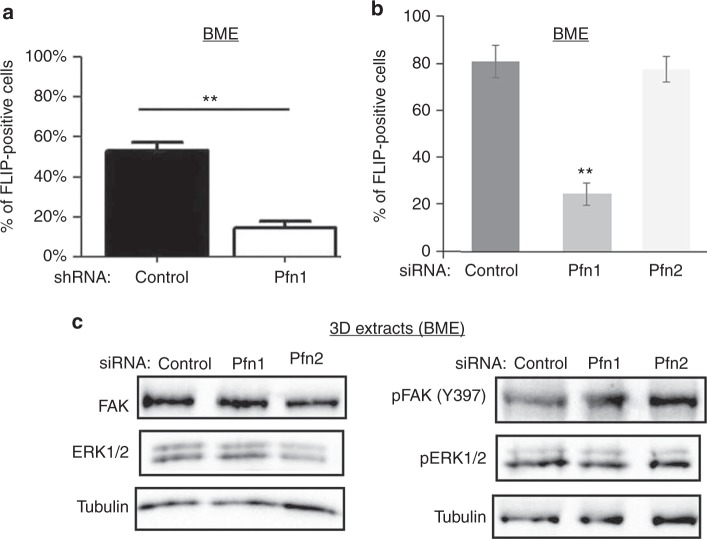
Effect of Pfn1 depletion on FLP formation, FAK, and ERK activation in MDA-231 cells. **a** Bar graph summarizing % FLP-positive cells in control- vs Pfn1-shRNA groups of cells in 3D BME-MoT assay. **b** Bar graph summarizing the changes in % FLP-positive cells upon isoform-specific depletion of Pfn by respective siRNA transfection. All FLP data are based on analyses of >60 cells in each group pooled from two independent experiments for each experimental setting; **p < 0.01). **c** Representative Immunoblot analyses of FAK, pFAK (Y397), ERK1/2, and pERK1/2 from 3D BME-MoT extracts of MDA-231 cells transfected with the indicated siRNAs (tubulin blot—loading control). Values are presented as mean ± SD

FLP induction has been shown to be a pre-requisite for outgrowth of BCC upon metastatic seeding. FLP enables the formation of cell-ECM adhesion plaques which then promotes dormancy-to-proliferation switch of BCC through activation of a FAK-Src-ERK signalling axis.^6,7^ Consistent with our outgrowth data, stable KD of Pfn1 significantly inhibited the FLP-forming ability of MDA-231 cells in MoT culture, as judged by the relative % of FLP-positive cells in control (~55%) vs Pfn1-KD (~10%) groups (Fig. 3a and supplementary Fig S5A). These findings were also reproducible in transient transfection setting where KD of Pfn1 but not Pfn2 dramatically reduced FLP formation in MDA-231 cells in MoT culture (Fig. 3b); although, the baseline values of % FLP-positive cells in both control and Pfn1 KD cells were higher in transient transfection setting. These data demonstrate that Pfn1 plays an important role in FLP formation in BCC.

**Fig. 4:**
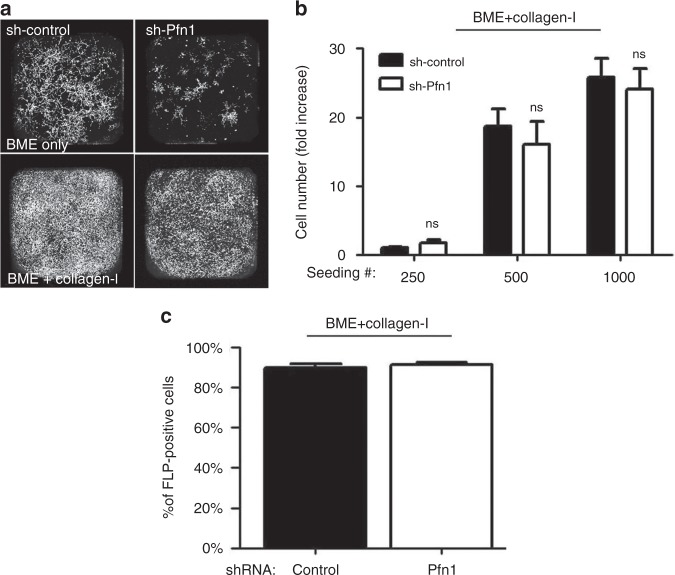
Collagen-I rescues FLP and outgrowth defects of Pfn1-deficient MDA-231 cells. **a** Representative images of outgrowth of control vs Pfn1-shRNA expressing MDA-231 cells on BME matrix with or without addition of collagen-I (images were taken on day 8 after seeding 500 cells/well). **b** A bar graph summarizing the final number of cells (counted on day 10) in control vs Pfn1 shRNA groups of MDA-231 cells seeded on collagen-I-supplemented BME matrix for different seeding densities (data summarised from two independent experiments with three technical replicates per experiment). **c** Quantification of % FLP-positive cells in control vs Pfn1-shRNA MDA-231 cells seeded on collagen-I-supplemented BME matrix. FLP data are based on analyses of >60 cells in each group pooled from two independent experiments for each experimental setting. Values are presented as mean ± SD

**Fig. 5:**
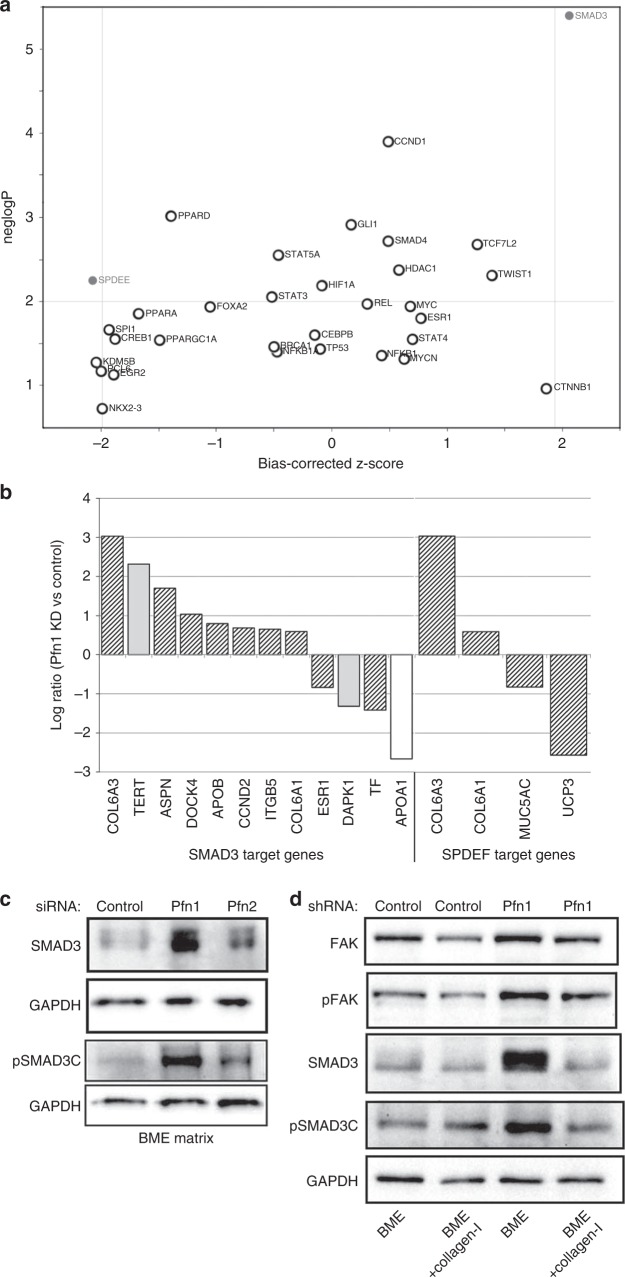
Analysis of predicted upstream regulators via ingenuity pathway analysis and biochemical confirmation. **a** Scatter plot depicting the predicted activation/inhibition status of upstream regulators belonging to the category of ‘transcription factors’. The *x*-axis represents the activation *z*-score, and the *y*-axis represents the negative logarithm of the significance of overlap between a regulator’s target genes and the differentially expressed genes in Pfn KD vs control samples. Regulators with an overlap *p*-value ≤0.01, and *z*-scores ≥2.0 or ≤−2.0 are considered to be significantly activated or inhibited, respectively. **b** List of target genes for the transcription factors SMAD3 and SPDEF, and their fold-change of expression (hatched bar: direction of gene changes consistent with predicted activation of SMAD3; grey bar: direction of gene changes not consistent; white bar: effects of direction of gene changes unknown). **c** Immunoblot analyses of SMAD3 and pSMAD3C from 3D BME-MoT extracts of MDA-231 cells transfected with the indicated siRNAs (GAPDH blot—loading control). **d** Immunoblot analyses of FAK, pFAK, SMAD3 and pSMAD3C from 3D extracts of control- vs Pfn1-shRNA expressing MDA-231 cells cultured in BME with or without collagen-I (data representative from three independent experiments)

Although, FLP defect has been causally linked to reduced FAK/ERK activation in BCC, surprisingly, immunoblot analyses of 3D BME-MoT culture extracts showed no discernible evidence of impairment in either the total protein expression or activation (as judged by the levels of phosphorylation) of FAK and ERK in MDA-231 cells, at least at a global level, upon transient KD of either Pfn1 or Pfn2 (Fig. 3c). Similarly, stable KD of Pfn1 also did not appear to attenuate the overall FAK activation in MDA-231 cells (Fig. 5d). It has been shown that stiffening of the microenvironment resulting from enhanced collagen-I deposition in the ECM during fibrosis can induce an outgrowth phenotype in otherwise dormant (non-proliferative) BCC.^2^ Outgrowth of BCC in fibrotic environment can be mimicked in MoT assays by addition of collagen-I to the BME matrix. Although Pfn1 KD does not appear to have any discernible effect on collagen-I expression per se in MDA-231 cells (supplementary Fig S6), addition of exogenous collagen-I dramatically stimulated the outgrowth of MDA-231 cells as expected and importantly completely rescued Pfn1 KD cells from their outgrowth deficiency (Fig. 4a, b). We also asked whether collagen-I-induced phenotypic switch was correlated with any change in the efficiency of FLP formation. Indeed, FLP formation was restored in stable Pfn1 KD cells in the presence of collagen-I (almost 100% of cells in either control or Pfn1-KD groups were found to FLP-positive upon collagen-I treatment) (Fig. 4c and supplementary Fig S5B). Overall, this strong correlation between FLP formation and outgrowth competency of BCC is consistent with a previous report.^7^ Collectively, these results suggest that Pfn1 depletion induces outgrowth defect in TNBC cells by affecting an ECM-stiffness sensitive signalling pathway that is important for BCC outgrowth, which is likely to be independent of the FAK/ERK signalling axis.

### Transcriptional regulation of canonical pathways and oncogenic gene expression signatures by Pfn1 KD

In order to gain further insights into the biological mechanisms that might underlie outgrowth defects of MDA-231 cells upon LOF of Pfn1, we initially performed differential gene expression analyses of control vs Pfn1 KD MDA-231 cells (two independent Pfn1 shRNA clones were analysed) in 2D culture settings and further carried out pathway enrichment and over-representation analyses from the microarray data. GSEA identified several canonical pathways that were significantly enriched for differentially expressed genes in Pfn1 KD samples (46 upregulated and 25 downregulated pathways at FDR ≤ 10%) (supplementary Table 1). To identify the key biological mechanisms captured by these pathways, we clustered the pathways based on similarities in gene-membership across the pathways (supplementary Fig S7). The results demonstrated pathways related to the broader themes of ECM organisation and G-protein signalling to be downregulated upon Pfn1 KD, whereas pathways affiliated to protein translation, inflammatory processes, DNA damage and p53 signalling were upregulated. In addition to the investigation of canonical pathways, we further used GSEA to interrogate gene expression patterns in Pfn1 KD that were significantly matched to curated oncogenic gene expression signatures available from MSigDB (C6: oncogenic signatures collection). At FDR ≤ 10%, we identified 2 gene expression signatures from JAK2 (Janus Kinase 2—a promoter of STAT signalling with oncogenic function) and PTEN (Phosphatase and Tensin homolog—an antagonist of PI3K signalling and a prominent tumour suppressor) KD studies that were significantly aligned to the Pfn1 KD transcriptome for both upregulated and downregulated genes. Specifically, the top 200 downregulated genes upon JAK2 knockdown in HEL cells (JAK2_DN.V1_DN) were significantly enriched in the Pfn1 KD downregulated genes (FDR = 0.002) whereas the top 200 upregulated genes from the same JAK2 KD study (JAK2_DN.V1_UP) showed significant overlap with the top Pfn1 KD upregulated genes (FDR = 0.068). Similarly, a significant proportion of genes highly downregulated upon PTEN KD (PTEN_DN.V1_DN) were also downregulated in Pfn1 KD (FDR = 0.096) cells, whereas genes upregulated in PTEN KD samples were also enriched for Pfn KD upregulated genes (FDR = 0.005). This is shown in supplementary Fig S7B, which display the GSEA enrichment plots for the Pfn1 KD gene expression data when compared to the oncogenic gene-set signatures for JAK2 and PTEN KD. Overall, these data demonstrate that Pfn1 downregulation can potentially impact oncogenic and tumour-suppressor pathways in BCC.

**Fig. 6:**
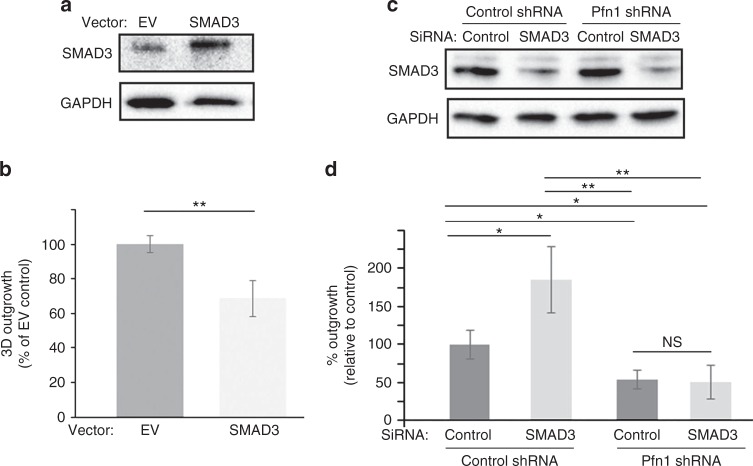
Effects of perturbation of SMAD3 on 3D outgrowth of MDA-231 cells in BME matrix. **a**–**b** **a:** SMAD3 and GAPDH (loading control) immunoblots of MDA-231 cells transfected with either empty vector (EV) control or SMAD3 overexpression vector (cells were transfected with 1 µg plasmid in 35 mm culture dish). Note that the SMAD3 immunoblot image was acquired at a very low exposure to avoid signal saturation of the overexpression band. **b** Summarises the outgrowth of SMAD3 overexpression group relative to EV control group of cells in BME matrix. **c**–**d** **c**: SMAD3 and GAPDH (loading control) immunoblots of total cell extracts of control and Pfn1-shRNA expressing MDA-231 cells transiently transfected with the indicated siRNAs. **d** Summarises the outgrowth of the various transfected groups relative to the control group (control shRNA expressers transfected with control siRNA) in BME matrix. Gene silencing and overexpression-based outgrowth data summarised from two independent experiments, with three technical replicates per experiment). *p < 0.05; **p < 0.01; NS not significant). Values are presented as mean ± SD

To further explore the candidate gene regulatory networks active in Pfn KD samples, we analysed Pfn KD responsive genes (absolute fold-change >1.5-fold) via the upstream regulator analysis (URA) function in Ingenuity. We identified 2 transcription factors, SMAD3 (a key mediator of TGFβ signalling) and SPDEF (an ETS family transcription factor that is required for tumourigenesis of certain subtypes of BC^25^ and transcriptionally repressed by SMAD3^26^) whose target genes showed significant overlap with Pfn KD responsive genes (*p*_overlap_ = 4.01E−06 for SMAD3, and 5.65E−03 for SPDEF, respectively) (Fig. 5a). The direction of gene expression changes in Pfn KD samples was largely consistent with a predicted activation of SMAD3 and inhibition of SPDEF (absolute *z*-score >2.0 for both regulators). Figure 5b demonstrates the transcriptomic changes observed upon Pfn KD in the gene-targets of SMAD3 and SPDEF. Additional details of URA are presented in Supplementary Table 2. SMAD3 is a central mediator of the tumour-suppressive function of TGFβ signalling. Furthermore, the tumour-suppressive function of SMAD3 is sensitive to ECM stiffness. Specifically, stiffening the ECM neutralises the antagonistic action of SMAD3 on cell proliferation.^27^ Given our findings that depletion of Pfn1 leads to outgrowth defect of BCC in BME matrix and this can be rescued by stiffening of ECM through addition of collagen-I, we asked whether these phenotypes are correlated with alterations in SMAD3 activation. Therefore, we performed immunoblot analyses of C-terminal phosphorylation of S423/S425 (pSMAD3C: this phosphorylation confers growth-inhibitory ability of SMAD3) as well as the total protein level of SMAD3 in 3D BME-MoT cultures of control vs Pfn1 KD MDA-231 cells. We found that consistent with the upregulation of gene expression signature of SMAD3, loss of Pfn1 in both transient and stable KD settings was associated with elevated levels of both total and phosphorylated SMAD3 (mean fold change >2.5-fold; *p* < 0.05) in MDA-231 cells in BME-MoT culture (note that Pfn2 KD did not elicit this effect). Addition of collagen-I to the MoT culture dramatically reduced the total and the phosphorylated levels of SMAD3 in Pfn1 KD cells normalizing their levels to those in control cells (Fig. 5c, d). The inverse correlation between SMAD3 and the outgrowth phenotype of Pfn1 KD MDA-231 cells in BME vs collagen-I-supplemented BME cultures prompted us to further probe whether could be a potential causal link between SMAD3 hyperactivation and outgrowth deficiency of BCC upon LOF of Pfn1. We first assessed the effect of SMAD3 overexpression on 3D outgrowth of MDA-231 cells in BME-MoT assay (cells transfected with the empty vector (EV) served as the control group). We optimised the transfection condition in pilot experiments to achieve overexpression-induced increase in SMAD3 (~3-fold increase) comparable to the level that is otherwise induced by Pfn1 KD (mean fold-increase: 3.2). Consistent with the tumour-suppressive function of SMAD3, transient overexpression of SMAD3 led to a significant 30% reduction in the 3D outgrowth of MDA-231 cells (Fig. 6a, b). Since these experiments were performed in a transient plasmid transfection setting where transfection efficiency of MDA-231 cells is typically in the range 60%, we wondered whether % inhibition of outgrowth upon SMAD3 overexpression is underestimated to some extent in our experimental settings. Therefore, we also performed outgrowth experiments with MDA-231 cells following infection with adenovirus encoding either EGFP (Ad-GFP)- or SMAD3 (Ad-Smad3). Although adenoviral infection resulted in ~80–90% of transduction efficiency (based on % GFP-positive cells) and a much more robust 6–7-fold SMAD3 upregulation, surprisingly, SMAD3 overexpression did not elicit a stronger suppression of single cell outgrowth of MDA-231 cells (supplementary Fig S8). The average % reduction of MDA-231 outgrowth upon Ad-SMAD3 infection (~20%), although statistically significant from Ad-GFP transduced group, was even slightly lower than what we saw in our plasmid transfection setting (possible reason for this is discussed later). Next, we assessed the effect of SMAD3 KD (using SMAD3 siRNA #1) on 3D outgrowth of control- vs Pfn1-shRNA transfected MDA-231 cells in BME-MoT assay. Immunoblot data based on cell lysates prepared in a parallel 2D culture showed dramatic ~80% downregulation of SMAD3 expression upon siRNA treatment (Fig. 6c). It is also important to note here that Pfn1-depletion induced upregulation of SMAD3 is only observed in 3D BME-MoT culture but not in rigid 2D tissue-culture environment, a data that is further consistent with collagen-I stiffened ECM-induced normalisation of SMAD3 between control and Pfn1 knockdown cells. In accord with our overexpression experiment results, control shRNA-bearing cells exhibited an increase in outgrowth in response to silencing of SMAD3; however, SMAD3-depletion alone was not sufficient to rescue the outgrowth-deficient phenotype of Pfn1-shRNA cells (Fig. 6d). To further verify these results with other siRNAs, we generated a tetracycline-inducible Pfn1 shRNA (this targeting sequence is also distinct from the constitutive Pfn1 shRNA and smart-pool Pfn1 siRNAs as described in previous experiments) expressing stable subline of MDA-231 cells through lentiviral infection and confirmed KD of Pfn1 upon doxycycline (dox) treatment (supplementary Fig S9A). As dox alone has inhibitory effect on cancer cell (breast, prostate and colorectal) growth,^28–30^ to avoid the confounding effect of dox on outgrowth, we independently assessed 3D outgrowth of untreated (represents Pfn1-proficient condition) and dox-treated (represents Pfn1-depleted condition) MDA-231 cells without or with SMAD3 KD using with a second siRNA (SMAD3 siRNA #2). Similar to our results shown in Fig. 6d, SMAD3 KD enhanced the outgrowth potential of untreated cells (supplementary Fig S9B-C) but did not have any statistically significant effect on the outgrowth readout of dox-treated cells (supplementary Fig S9D-E). Collectively, the results from SMAD3 perturbation experiments suggest that while SMAD3 upregulation can potentially partly contribute to suppression of outgrowth, additional proliferation-restraining mechanisms are likely operative in conjunction with SMAD3 to account for the outgrowth-deficient phenotype of BCC cells upon depletion of Pfn1.

## Discussion

In this study, we demonstrated that Pfn1 deficiency significantly impairs FLP forming ability and single cell outgrowth competency of BCC. In accord with these data, clinical correlation findings further revealed lower Pfn1 expression associated with lower nuclear grade tumours and longer RFS of BC patients. While reduced FLP abundance and outgrowth deficiency of BCC upon Pfn1 KD is consistent with previously reported LOF phenotypes of other Pfn1-binding actin-cytoskeleton regulatory proteins (such as mDia2 and Ena/VASP),^6,7^ and may even indirectly suggest possible role of Pfn1’s interaction with those actin-binding proteins, in this context, there is an important distinction between the previous and our findings. Although impaired FAK/ERK activation resulting from FLP defect has been causally linked to outgrowth deficiency of BCC upon LOF of various actin-cytoskeleton regulatory proteins, surprisingly, this does not appear to be true in the case of Pfn1. This might suggest that FAK/ERK activation may be promoted by but is not absolutely dependent on FLP induction in BCC. Furthermore, physiological levels of FAK/ERK signalling may be necessary but not sufficient to drive efficient outgrowth of BCC as it has been postulated in the literature based on the experimental findings involving overexpression of constitutively active FAK construct (which likely does not mimic a physiological scenario).

In this study, we showed that loss of Pfn1 leads to SMAD3 upregulation in BCC when cultured in soft ECM environment (such as BME matrix) but this effect is abrogated when cells are cultured in a stiffer environment (i.e. either rigid tissue-culture plate or collagen-I-stiffened BME matrix). Interestingly, a recent study has shown that Pfn2 can promote SMAD3 transcription epigenetically in lung cancer cells through suppressing the recruitment of HDAC1 to the SMAD3 promoter.^14^ In our case, we do not believe that loss of Pfn1 is promoting SMAD3 transcription indirectly through affecting Pfn2 activity (possibly through relieving a competition between two isoforms for a common binding factor) for two reasons. First, we did not see any appreciable change in either SMAD3 and phospho-SMAD3 level in MDA-231 cells upon Pfn2 KD. Second, our microarray data did not reveal any change in the SMAD3 mRNA level upon Pfn1 KD, it is possible that SMAD3 is not transcriptionally regulated (however since our microarray data were based on 2D cell culture, RT-PCR experiments from 3D cultures will be necessary to more definitively establish transcriptional vs post-transcriptional upregulation of SMAD3 in Pfn1-deficient condition). It has been shown that SMAD3 level can be post-translationally regulated through protein-stability control via phosphorylation of certain residues (S245, S250, and S255) in its linker region (phosphorylation of these residues increases half-life of SMAD3). Therefore, it will be interesting to further explore whether loss of Pfn1 somehow impacts protein stability of SMAD3 through modulation of its linker phosphorylation, and there is any dependence on substrate stiffness for this event. MAP kinases (ERK, JNK) have been shown to promote linker phosphorylation of SMAD3.^31,32^ As we did not find any evidence of enhanced ERK activation in response to Pfn1 KD, it is unlikely that SMAD3 upregulation in Pfn1 KD cells is ERK signalling dependent; however, potential role of other members of MAP kinase family cannot be ruled out.

Our studies clearly suggest that there are other proliferation-restraining signals which can dominate over proliferation-inducing effect of FAK/ERK signalling. Specifically, in the context of Pfn1, based on collagen-I’s ability to concomitantly relieve Pfn1-deficient cells from SMAD3 hyperactivation (likely a direct result of SMAD3 upregulation) and outgrowth-arrested phenotype, probing a possible causal relationship between hyperactive SMAD3 signalling and outgrowth-deficient phenotype upon Pfn1 depletion was clearly a logical step in our study. Although previous studies have shown that SMAD3 can promote FLP in breast (including MDA-231) and gastric cancer cells (an effect that is indirectly related to SMAD3’s ability to transcriptionally upregulate actin-cross-linking protein fascin, an important molecular player of filopodial assembly),^33,34^ our MoT-outgrowth experiments in SMAD3 overexpression and knockdown settings demonstrated an inhibitory effect of SMAD3 on single cell 3D outgrowth of BCC. While these data are generally consistent with the tumour-suppressive functionality of SMAD3, the relatively modest effect of SMAD3 overexpression on single cell 3D outgrowth can be potentially explained by the possibility of the FLP-promoting effect (an outgrowth-promoting feature in single-cell outgrowth setting) partly negating the anti-proliferative action of SMAD3. In fact, our preliminary observations of reduced abundance of FLP in MDA-231 cells in MoT assay upon SMAD3 KD (supplementary Fig S10) is consistent with this possibility. Given that SMAD3 silencing was not sufficient to reverse the outgrowth-deficient phenotype of Pfn1-deficient cells suggesting that additional brakes (likely ECM stiffness-sensitive) in conjunction with SMAD3 are operative in Pfn1-deficient cells to restrain their proliferative capacity. Our gene-set enrichment analyses suggested upregulation of p53 (tumour-suppressor) and downregulation of JAK2 (oncogenic) and PTEN (tumour-suppressor) signalling pathways upon Pfn1 KD. Among these three pathways, upregulation of p53 and downregulation of JAK2 signalling are consistent with reduced outgrowth of BCC conferred by Pfn1 KD. Although downregulation of PTEN signalling is not consistent with the outgrowth phenotype of Pfn1 KD cells, it correlates with our previously published findings of Pfn1’s ability to negatively regulate PI3K-generated lipid products (PI(3,4)P_2_ and PIP_3_) and AKT signalling in MDA-231 cells.^35,36^ A recent study has shown that p53 activation in BCC is substrate stiffness-sensitive and that p53 elevation can suppress FLP.^37^ Since it was shown that softer substrate actually represses p53 activation, it is somewhat unlikely that modulation of p53 pathway accounts for collagen-I-induced reversal of outgrowth-deficient phenotype of Pfn1-depleted cells. Therefore, unbiased proteomic analyses of cellular extracts prepared in BME vs collagen-I-supplemented BME matrix culture environments will be necessary to identify Pfn1- and stiffness-sensitive proliferation-regulatory pathways for further mechanistic studies in the future.

Mortality of BC patients primarily results from overt metastatic growth from disseminated cancer cells in the distant organs. Therefore, molecular strategies that could induce dormancy-like phenotype in BCC may lead to a conceptual framework of new lines of targeted therapy against metastatic BC. We previously showed that loss-of-Pfn1 expression promotes dissemination but impairs metastatic colonisation ability of MDA-231 BCC in the lungs.^10^ The dichotomous effect of Pfn1 on early vs late steps of BC metastasis could be a possible reason why the OS (affected by both dissemination and growth of BCC) of BC patients has no significant association with Pfn1 expression at least at the gene expression level. Outgrowth-deficient phenotype of MDA-231 cells in BME matrix (closely approximates the mechanical compliance of lungs) upon Pfn1 KD suggest that metastatic colonisation defect of these cells in the lungs as shown previously our group can be a result of reduced proliferation and outgrowth. Pfn1 dependency for efficient outgrowth of BCC also seems to be consistent with lower Pfn1 expression correlated with longer RFS (dictated primarily by outgrowth) of BC patients. Therefore Pfn1 may be a novel biomarker for BC recurrence and a possible target to reduce metastatic outgrowth of BCC. However, a more comprehensive study with different molecular subtypes of BCC supported by in vivo studies is needed to fully justify this conclusion.

Finally, our studies show that outgrowth-deficiency of BCC upon Pfn1 depletion is cell-density dependent and rescuable by collagen-I. Therefore, it is possible that Pfn1 depletion may become an ineffective strategy to halt metastatic outgrowth of BCC in certain microenvironment (such as a stiffer organ or possibly under fibrotic condition in otherwise a soft organ) or at an advanced stage of the disease when too many micro-metastases have already developed. Future in vivo experiments are needed to address these outstanding questions.

## Electronic supplementary material

Supplementary material

Fig S1

Fig S2

Fig S3

Fig S4

Fig S5

Fig S6

Fig S7

Fig S8

Fig S9

Fig S10

Supplementary table S1

Supplementary table S2

## Availability of data and materials

Gene-specific survival outcomes in cancer patients were obtained through kmplot.com, a public domain data analysis tool that utilises curated genomic and clinical data available through Gene Expression Omnibus (GEO), European Genome-Phenome Archive (EGA) and The Cancer Gene Atlas (TCGA).
