# Supplementary material for: Profilin-1 deficiency leads to SMAD3 upregulation and impaired 3D outgrowth of breast cancer cells
Source: Br J Cancer. 2018 Oct 15;119(9):1106–17. doi: 10.1038/s41416-018-0284-6 (PMC6219497; doi:10.1038/s41416-018-0284-6)
Supplement: Supplementary file 1 — Supplementary material [file 41416_2018_284_MOESM1_ESM.docx]

**Supplemental Figures and Tables**

**Figure S1: Association between isoform-specific Pfn expression and overall survival (OS) of BC patients.** Kaplan-Meier survival plots showing the effect of Pfn1 and Pfn2 gene expression (above or below median levels of expression) on the OS based on analyses of transcriptome datasets for BC tumors (1402 samples) using *kmplot.com*. Note that compared to RFS data, OS data were available for less number of patients.

**Figure S2: Effect of Pfn1 depletion on MDA-231 growth in 2D monolayer culture.** Proliferation kinetics of parental, control - and Pfn1 shRNA cells on 2D tissue-culture dish (equal number of cells in each group were plated overnight in triplicates, serum-starved for 24 hours and then allowed to proliferate in serum-containing media for another 48 hours). These data are summarized from 3 independent experiments (*p < 0.05).

**Figure S3.** **Effect of Pfn1 depletion on the kinetics of MDA-231 outgrowth on 3D BME matrix.** Outgrowth kinetics of control and Pfn1 KD MDA-231 cells on BME matrix at different cell-seeding densities (250, 500, 1000 cells/well). These data are based on real-time cell count analyses of 3 technical replicates/experimental condition from 2 independent experiments). Values are presented as mean ± SD.

**Figure S4: Ectopic expression of GFP-Pfn1 enhances the outgrowth proficiency of Pfn1-deficient MDA-231 cells. (A)** Immunoblots of GFP-Pfn1, endogenous Pfn1 and tubulin (loading control) from total extracts of GFP-Pfn1-expressing subline of MDA-231 cells transfected with either a single-target (ST) Pfn1 siRNA (silences only endogenous Pfn1) or smart-pool (SP) Pfn1 siRNA (silences both endogenous Pfn1 and exogenous GFP-Pfn1); cells transfected with control siRNA served as a group for comparison. **(B)** A bar graph comparing the 3D outgrowth of GFP-Pfn1-expressors in BME matrix between ST- vs SP-Pfn1 siRNA transfection conditions (data summarized from 2 independent experiments for 3 technical replicates/experimental groups). Values are presented as mean ± SD.

**Figure S5: Matrix composition affects FLP-induction capability of Pfn1-depleted MDA-231 cells** Representative images of phalloidin (green) and DAPI (blue) staining of control and Pfn1-shRNA MDA-231 cells 3 days after seeding on BME matrix without (*panel A*) or with addition of collagen-I (*panel B*). Arrows show FLP (identified by phalloidin staining).

**Figure S6:** **Silencing Pfn1 does not affect collagen-I expression in MDA-231 cells**. **(A)** Representative images of immunostaining of Collagen I (green) in control- vs Pfn1-shRNA expressing MDA231 cells (nuclei (blue) were counterstained with DAPI). Cells stained with secondary antibody only (shown alongside) were used as negative control and for background intensity correction. **(B)** A bar graph summarizing the average intensity of Collagen I staining of Pfn1-shRNA expressing cells relative to that of control cells (data based on analyses of 150-200 cells pooled from two independent experiments for each experimental condition; ns: not significant).

**Figure S7.** **Gene-set enrichment analysis (GSEA) of Pfn KD vs. control samples.** **(A)** Pathways significantly enriched for differentially expressed genes in MDA-231 cells upon Pfn1 KD were visualized as a gene-overlap based clustered network, via the Enrichment map tool in Cytoscape. Gene-set clusters corresponding to broad functional categories are circled. Upregulated gene-sets are shown in red and down-regulated gene-sets in blue. **(B)** GSEA derived enrichment plots for oncogenic signature gene-sets for JAK2 and PTEN, in Pfn KD vs control samples. Gene expression observed in Pfn KD vs. control samples was consistent with an upregulation of genes typically downregulated upon knockdown of JAK2 or PTEN. Conversely, genes downregulated in Pfn KD samples display significant overlap with genes that show increased expression upon JAK2 or PTEN knockdown. The relative ranks of JAK2 or PTEN2 signature genes (based on their expression in Pfn KD and control samples) are indicated by the position of the lines at the bottom of the plot. Genes are ranked in descending order, based on their fold-difference between Pfn KD and controls with positive fold-changes shown in shades of red and negative fold-changes in shades of blue.

**Figure S8: Effect of adenoviral mediated SMAD3 overexpression on MDA-231 outgrowth. (A)** SMAD3 immunoblot of MDA-231 cell lysates 72 hrs after transduction of either Ad-GFP (control) or Ad-SMAD3 (GAPDH blot serves as the loading control). **(B)** A bar graph comparing the 3D outgrowth of Ad-SMAD3 transduced cells relative to Ad-GFP transduced cells 8 days after seeding on BME matrix (data summarized from 2 independent experiments for 8 technical replicates/experimental group; **: p<0.01).

**Figure S9: Confirmation of the finding that SMAD3 depletion alone fails to rescue Pfn1 KD cells from their outgrowth defect using an additional set of SMAD3 siRNA (siRNA set #2). (A)** Pfn1 and GAPDH (loading control) immunoblots of MDA-231 cells stably expressing dox-inducible Pfn1-shRNA demonstrate Pfn1 KD 3 days after dox treatment. (**B-E**) SMAD3 and GAPDH (loading control) immunoblots (*panels B, D*) of untreated vs dox-treated cells following transfection of either control or SMAD3 siRNA (siRNA #2). Bar graphs in panel C and E show outgrowth of untreated and dox-treated cells treated with the indicated siRNA relative to the respective control 7 days after seeding (data summarized from 2 independent experiments for 8 technical replicates/experimental group; **: p<0.01; NS: not significant).

**Figure S10: Effect of SMAD3 KD on FLP in MDA-231 cells in MoT assay.** Representative phase-contrast images of control and SMAD3 KD (using siRNA #2) MDA-231 cells 2 days after seeding on BME matrix (arrows show FLP; scale bar – 50 μm).

**Supplementary Table 1: Significantly up- and down-regulated pathways identified through Gene Set Enrichment Analysis (GSEA).** Canonical pathways and pathways composed of oncogenic signatures were obtained from MSigDB. Pathways with a false discovery rate (FDR) <10% are listed.

**Supplementary Table 2: Upstream Regulator Analysis in Ingenuity Pathway Analysis.** Regulators with an absolute bias-corrected z-score>1.5 are shown. Col 1, name of upstream regulator; col 2, log ratio of expression for upstream regulator in current study (only listed in absolute log ratio >0.58); col 3, type of upstream regulator; col 4, predicted state of upstream regulator (based on absolute value of activation z-score >2); col 5, activation z-score values; col 6, bias term value; col 7, bias-corrected z-score; col 8, p-value for overlap of list contents with regulator targets in IPA knowledgebase; col 9, list of target genes in experimental list.
