# Supplementary figures and images for: Profilin-1 deficiency leads to SMAD3 upregulation and impaired 3D outgrowth of breast cancer cells
Source: Br J Cancer. 2018 Oct 15;119(9):1106–17. doi: 10.1038/s41416-018-0284-6 (PMC6219497; doi:10.1038/s41416-018-0284-6)

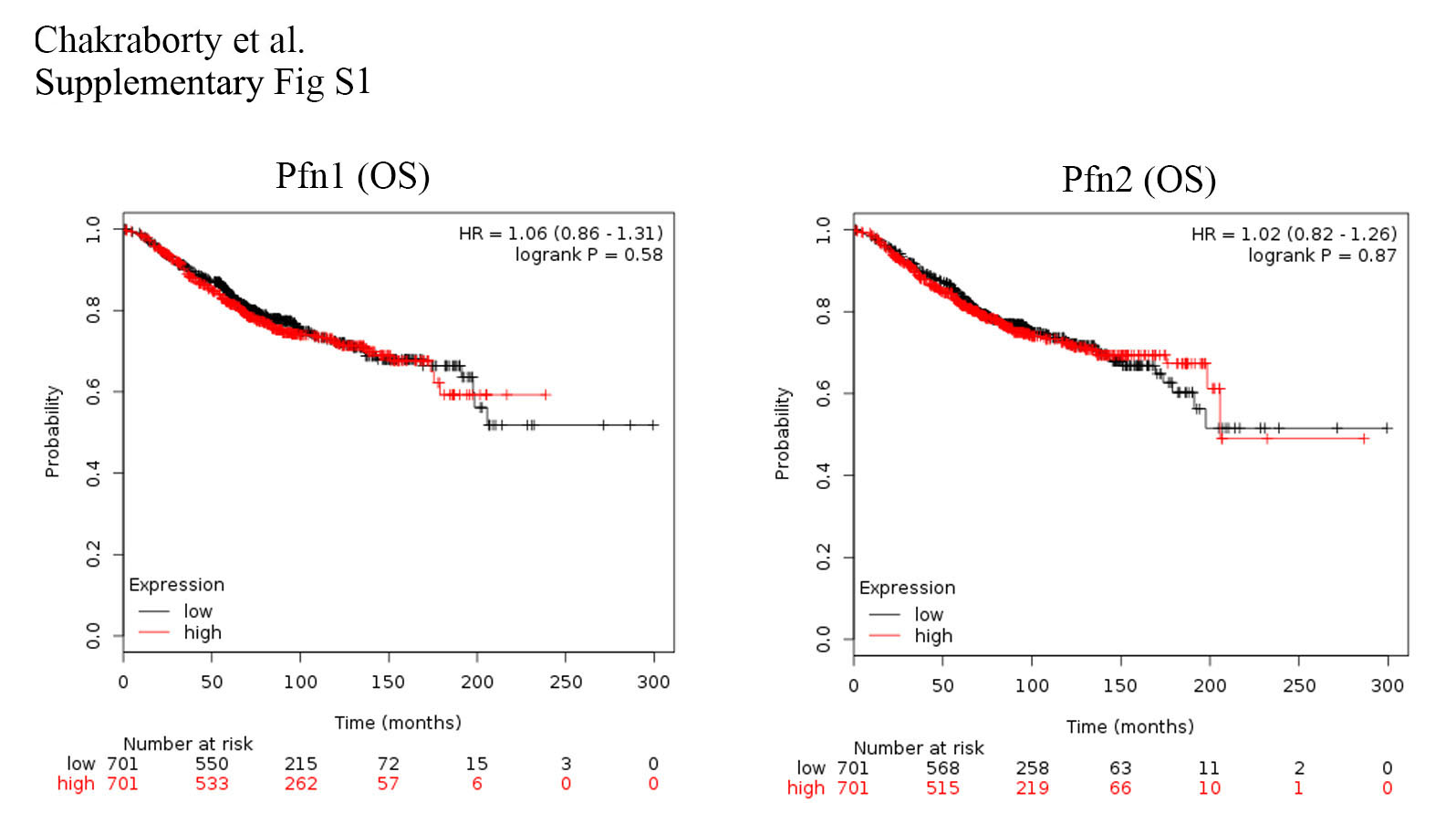

Supplement: Supplementary file 2 — Fig S1 [file 41416_2018_284_MOESM2_ESM.tif]

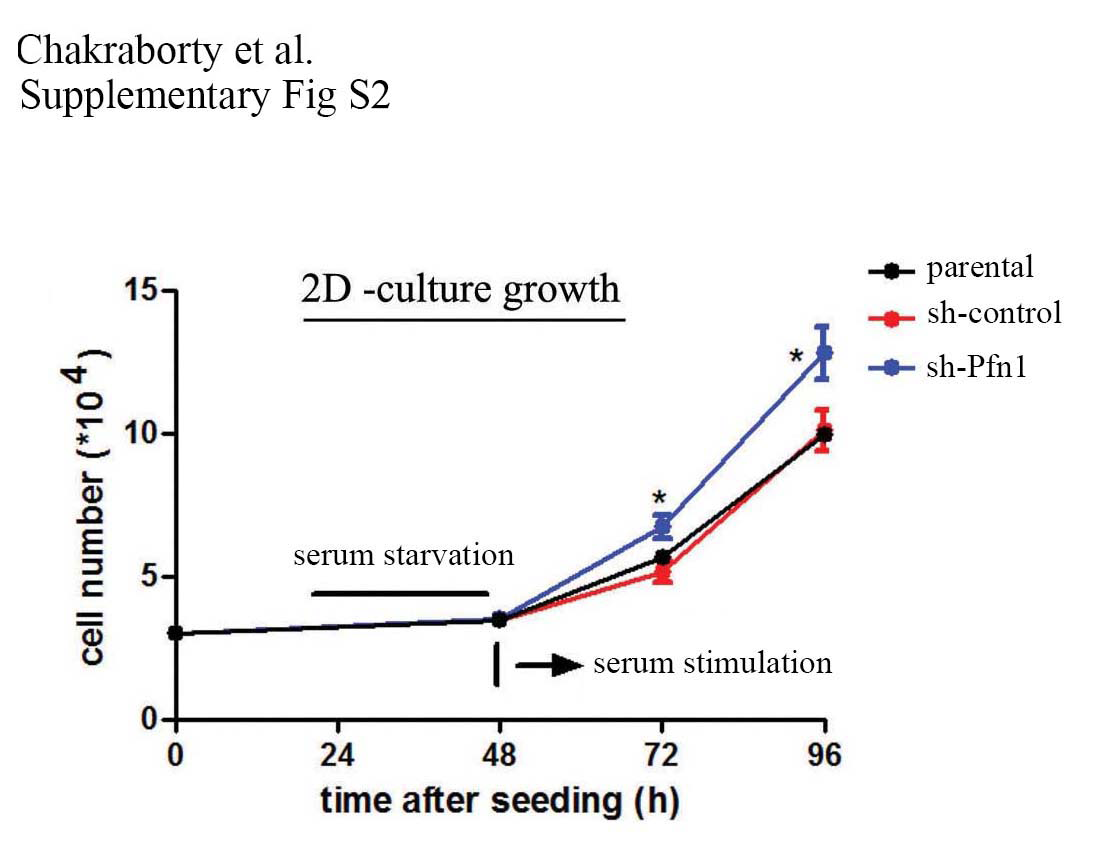

Supplement: Supplementary file 3 — Fig S2 [file 41416_2018_284_MOESM3_ESM.tif]

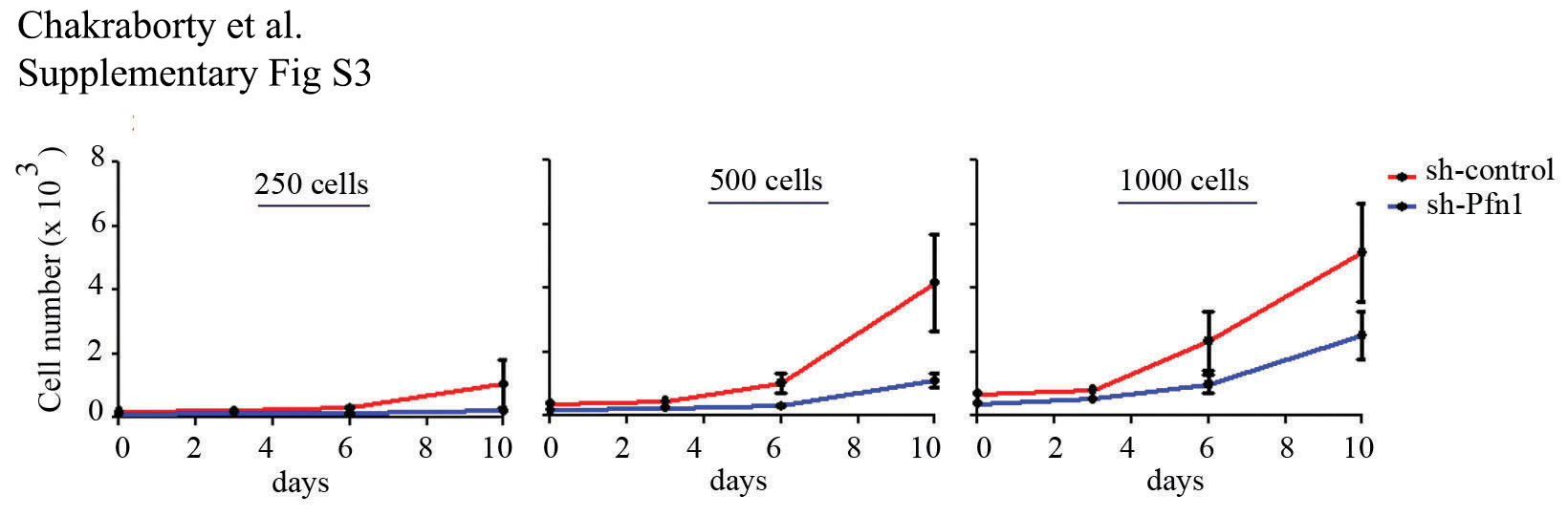

Supplement: Supplementary file 4 — Fig S3 [file 41416_2018_284_MOESM4_ESM.tif]

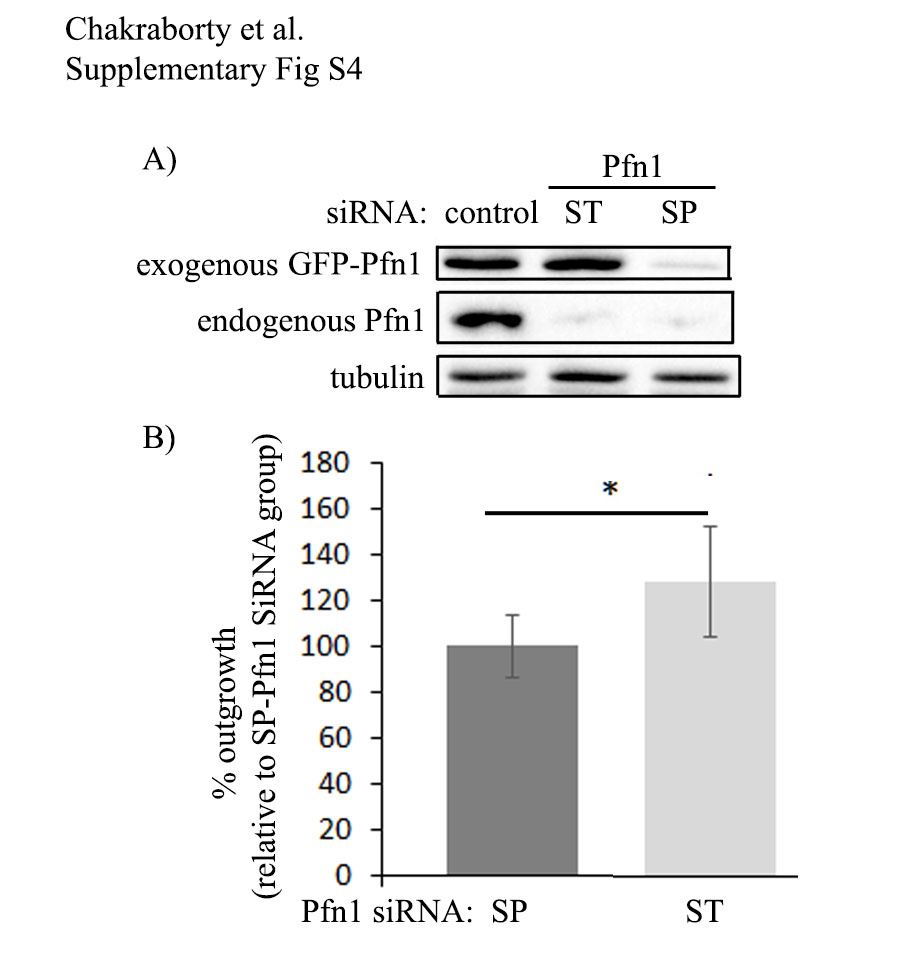

Supplement: Supplementary file 5 — Fig S4 [file 41416_2018_284_MOESM5_ESM.tif]

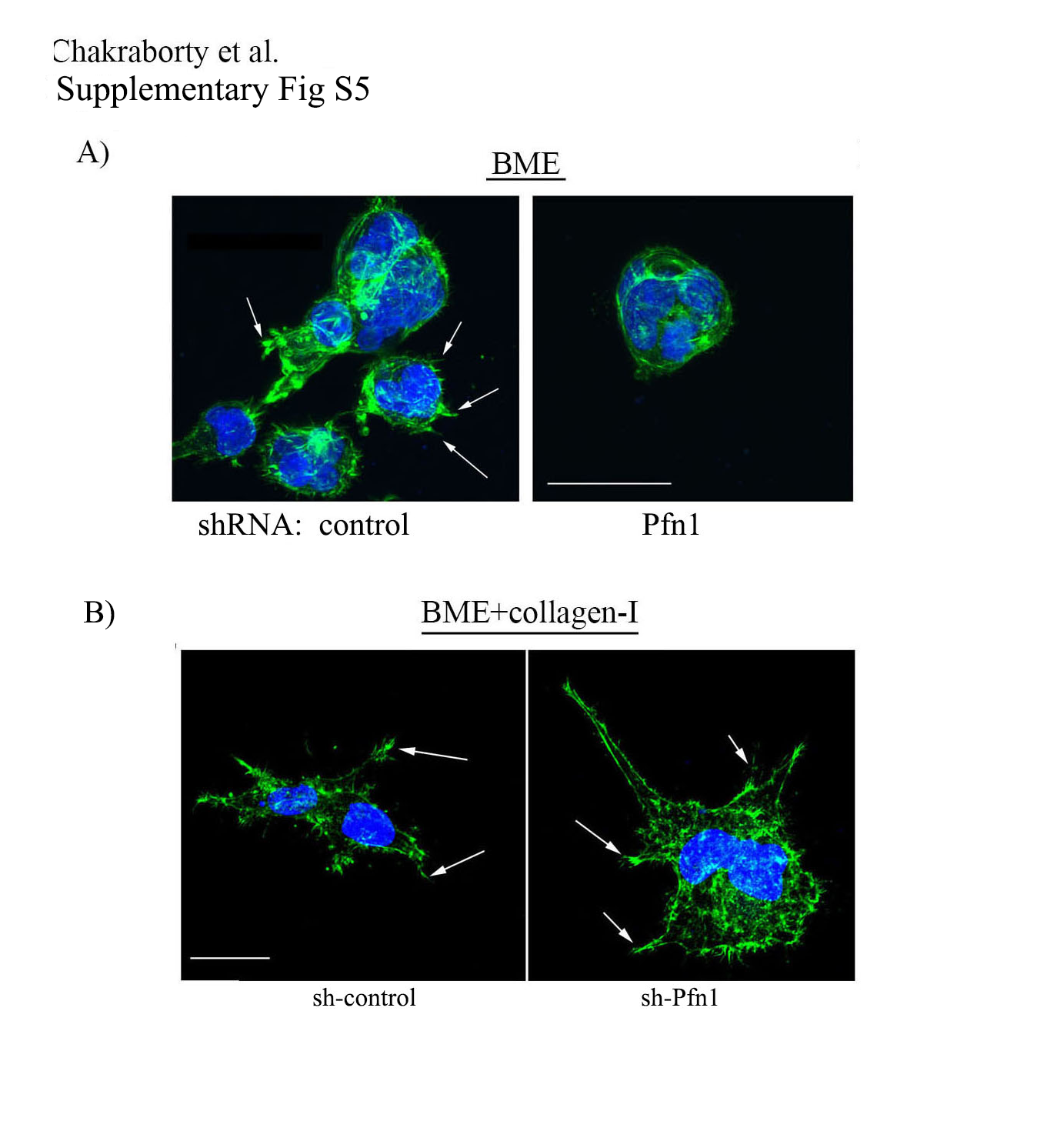

Supplement: Supplementary file 6 — Fig S5 [file 41416_2018_284_MOESM6_ESM.tif]

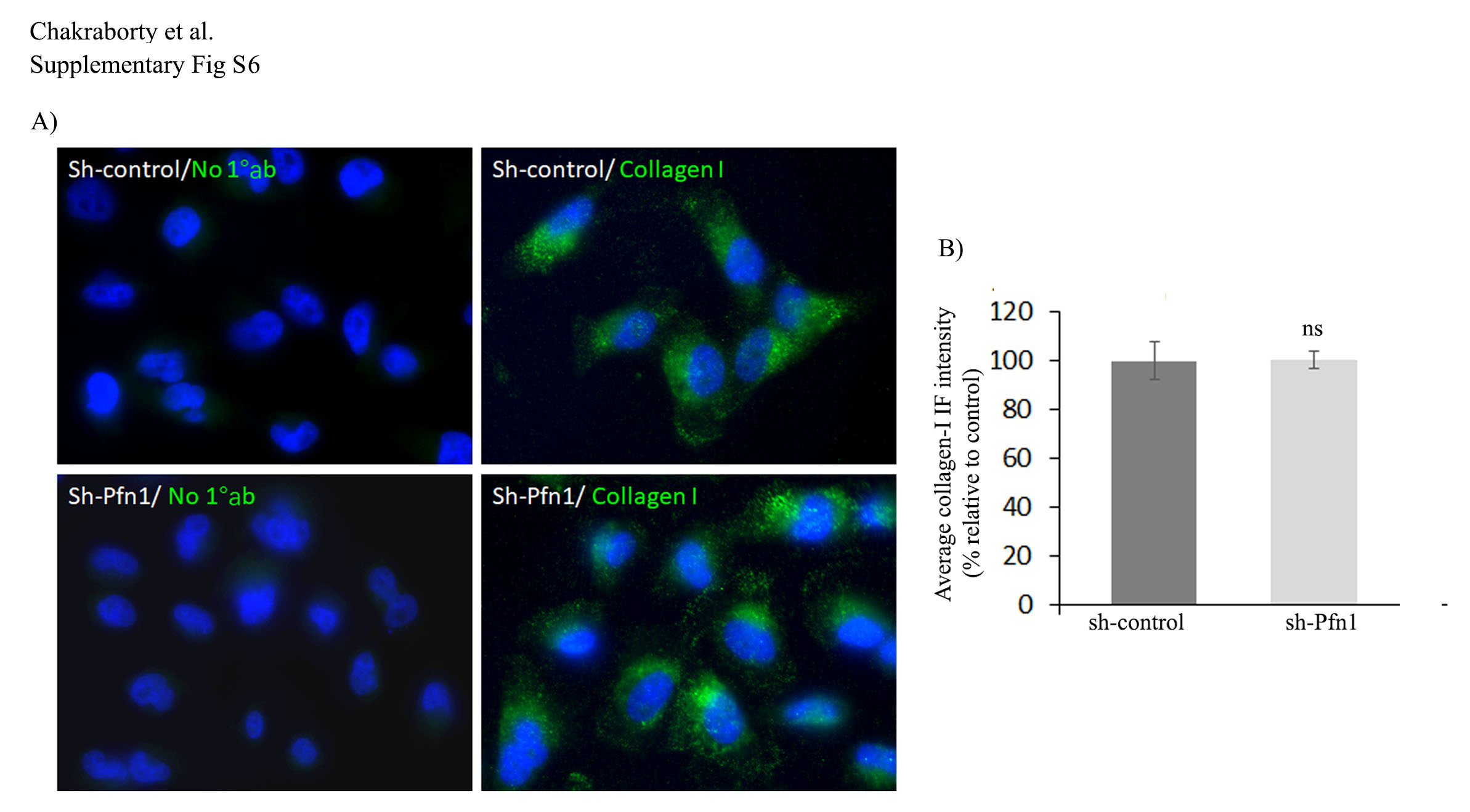

Supplement: Supplementary file 7 — Fig S6 [file 41416_2018_284_MOESM7_ESM.tif]

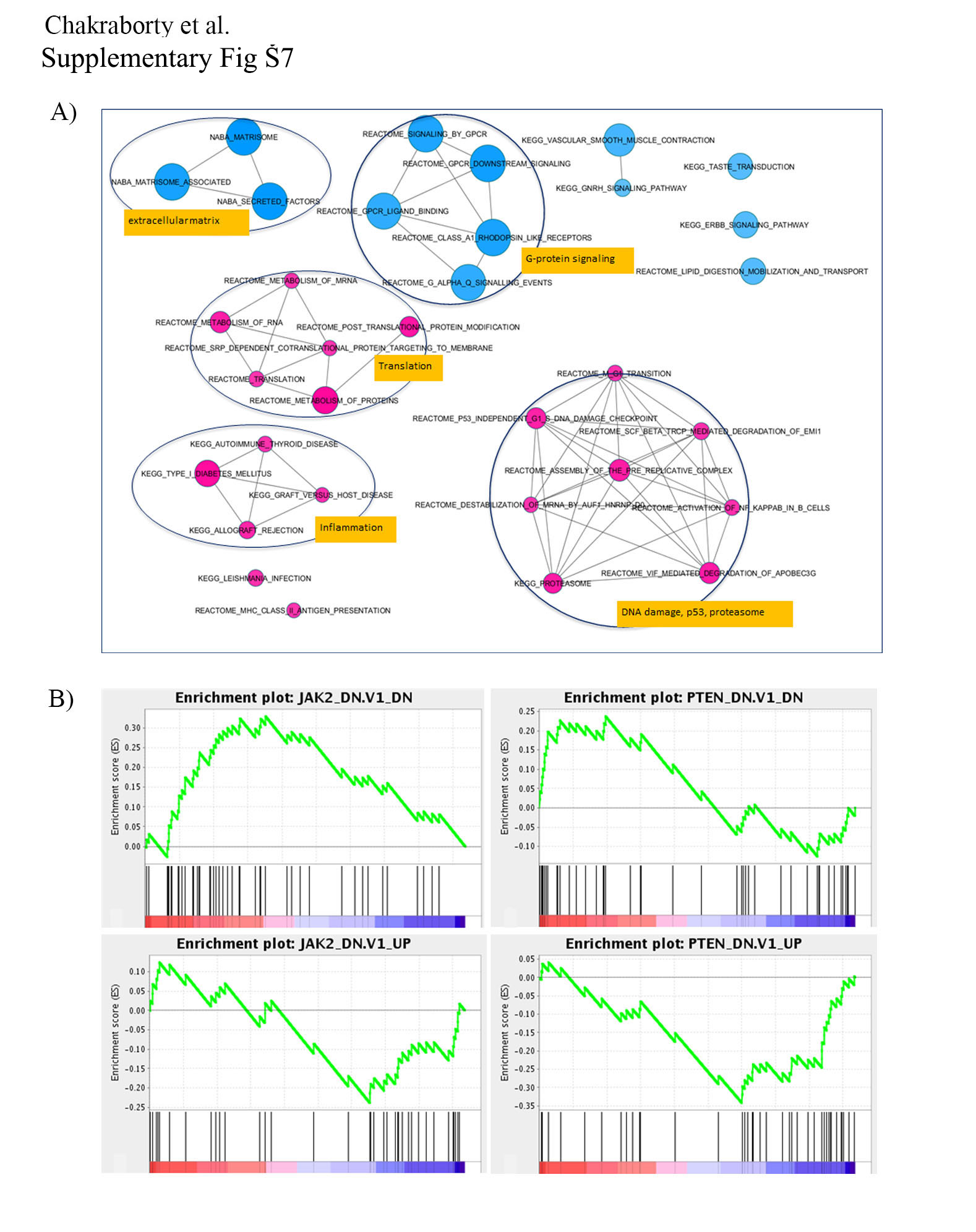

Supplement: Supplementary file 8 — Fig S7 [file 41416_2018_284_MOESM8_ESM.tif]

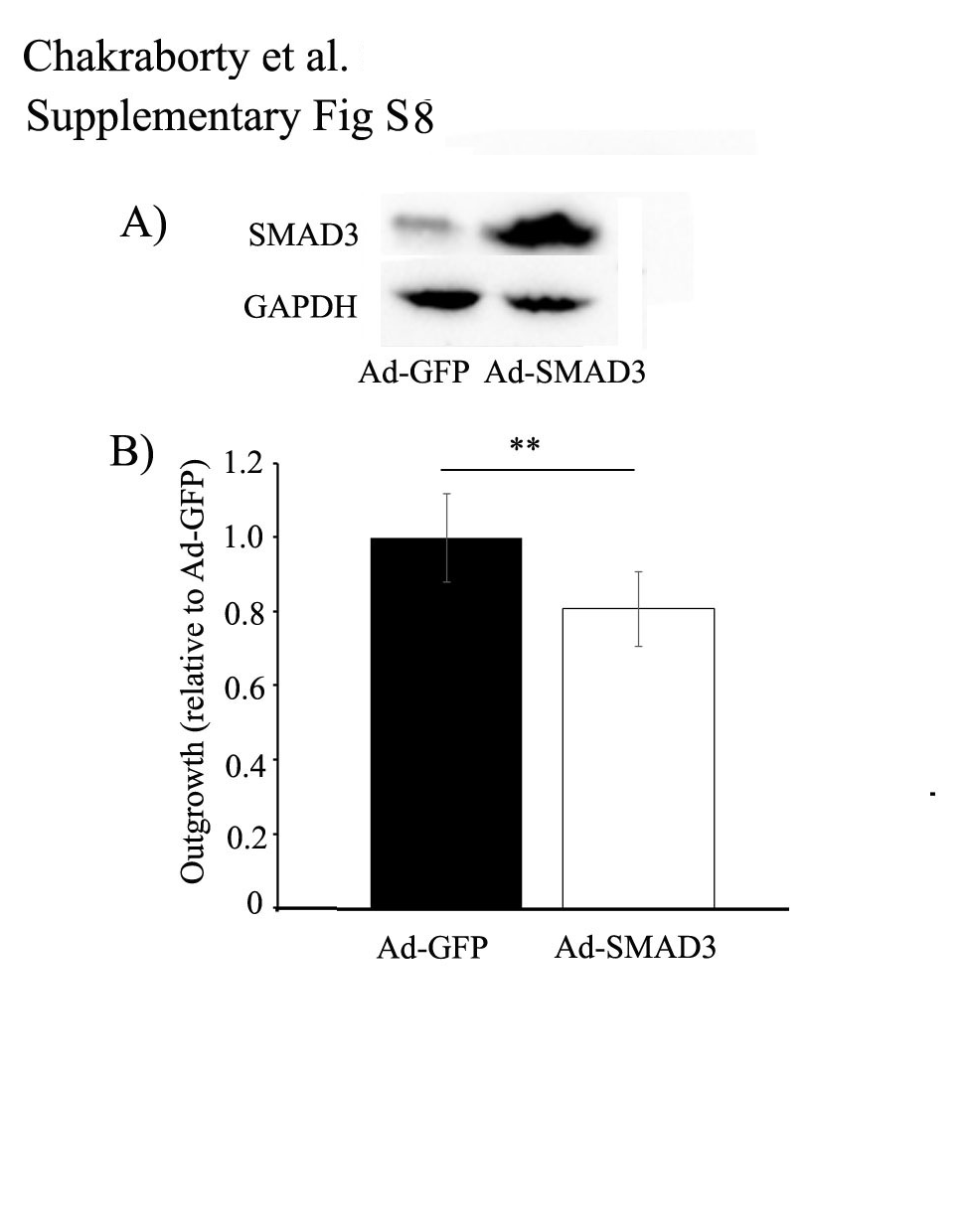

Supplement: Supplementary file 9 — Fig S8 [file 41416_2018_284_MOESM9_ESM.tif]

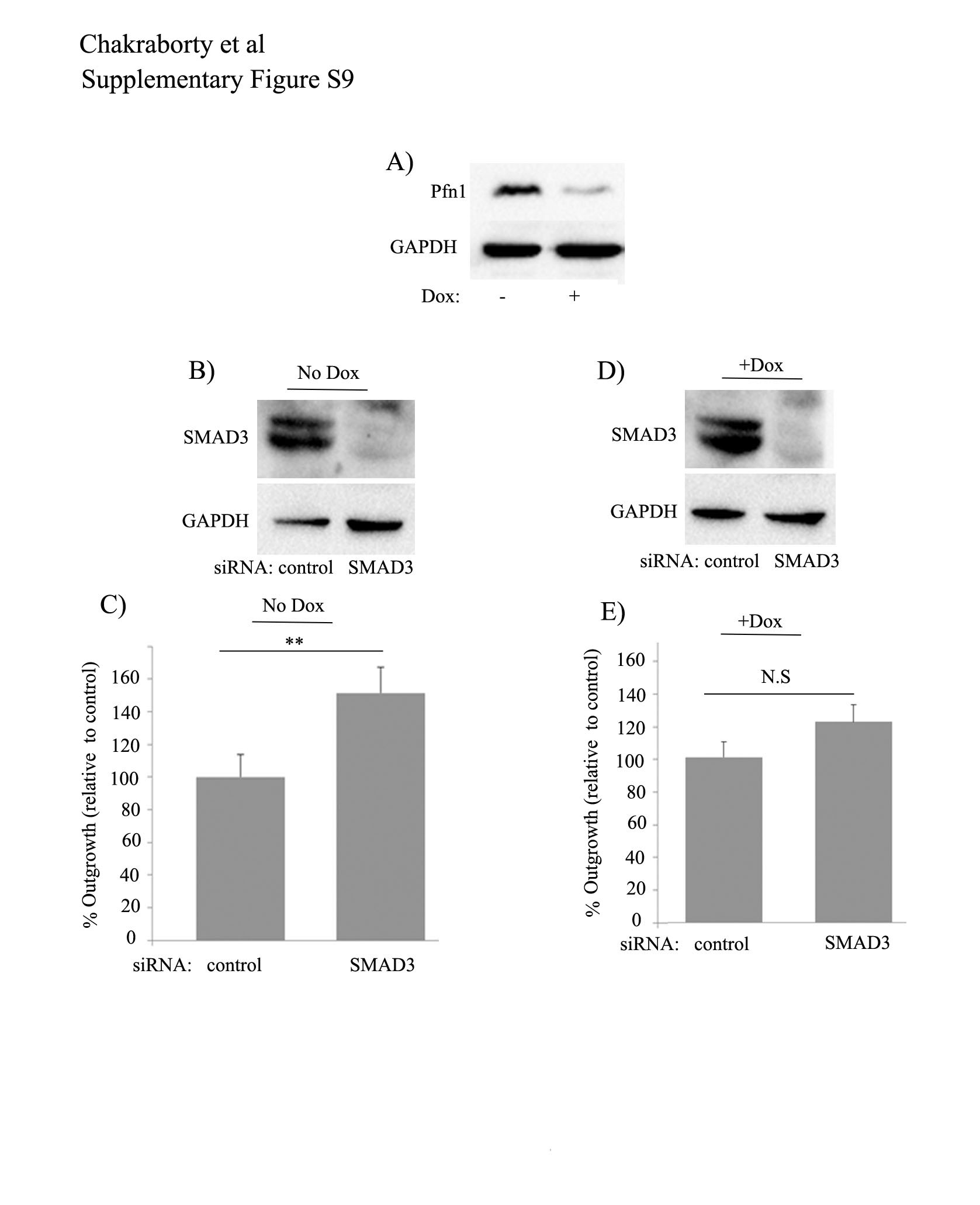

Supplement: Supplementary file 10 — Fig S9 [file 41416_2018_284_MOESM10_ESM.tif]

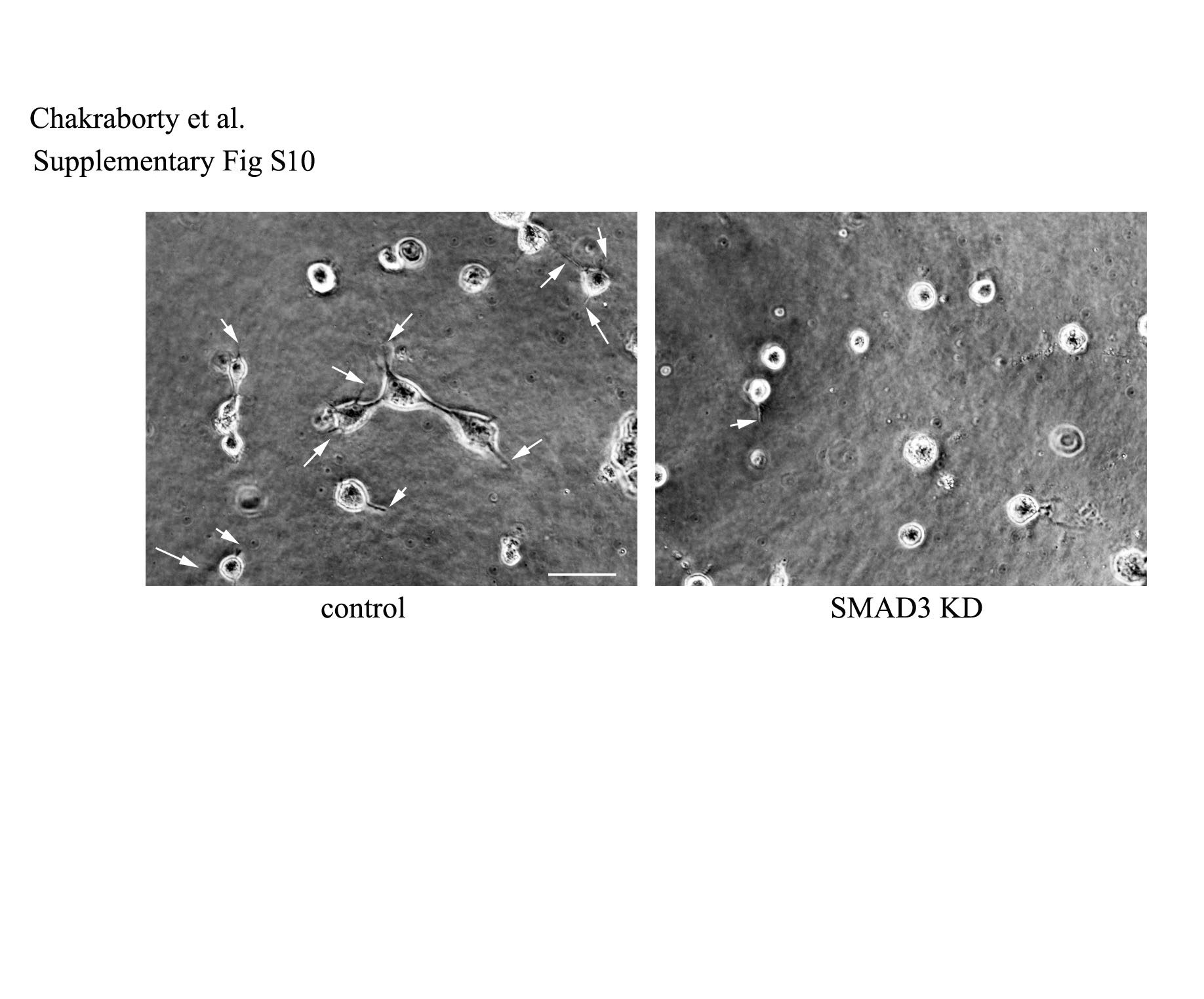

Supplement: Supplementary file 11 — Fig S10 [file 41416_2018_284_MOESM11_ESM.tif]
