# Supplementary material for: Profilin-1 deficiency leads to SMAD3 upregulation and impaired 3D outgrowth of breast cancer cells
Source: Br J Cancer. 2018 Oct 15;119(9):1106–17. doi: 10.1038/s41416-018-0284-6 (PMC6219497; doi:10.1038/s41416-018-0284-6)
Supplement: Supplementary file 12 — Supplementary table S1 [file 41416_2018_284_MOESM12_ESM.pdf]

SUPPLEMENTARY TABLE 1

NAME	SIZE	ES	NES	NOM p-val	FDR q-val	Treatment	Pathway
NABA_MATRISOME	190	-0.20944	-3.32122	0.0000	0.0000	Pfn1vscontrol_dnPfn1	msigdb_canonical
REACTOME_GPCR_DOWNSTREAM_SIGNALING	123	-0.24465	-3.1736	0.0000	0.0000	Pfn1vscontrol_dnPfn1	msigdb_canonical
NABA_SECRETED_FACTORS	63	-0.33773	-3.14725	0.0000	0.0000	Pfn1vscontrol_dnPfn1	msigdb_canonical
REACTOME_CLASS_A1_RHODOPSIN_LIKE_RECEPTORS	54	-0.34565	-3.01704	0.0000	0.0003	Pfn1vscontrol_dnPfn1	msigdb_canonical
REACTOME_SIGNALING_BY_GPCR	149	-0.20654	-2.95743	0.0000	0.0007	Pfn1vscontrol_dnPfn1	msigdb_canonical
REACTOME_PEPTIDE_LIGAND_BINDING_RECEPTORS	29	-0.45189	-2.84761	0.0000	0.0008	Pfn1vscontrol_dnPfn1	msigdb_canonical
NABA_MATRISOME_ASSOCIATED	139	-0.21547	-2.84374	0.0000	0.0008	Pfn1vscontrol_dnPfn1	msigdb_canonical
REACTOME_G_ALPHA_Q_SIGNALLING_EVENTS	41	-0.36414	-2.75951	0.0000	0.0013	Pfn1vscontrol_dnPfn1	msigdb_canonical
REACTOME_GPCR_LIGAND_BINDING	75	-0.2737	-2.72658	0.0000	0.0014	Pfn1vscontrol_dnPfn1	msigdb_canonical
KEGG_CYTOKINE_CYTOKINE_RECEPTOR_INTERACTION	56	-0.283	-2.41797	0.0000	0.0103	Pfn1vscontrol_dnPfn1	msigdb_canonical
KEGG_CALCIUM_SIGNALING_PATHWAY	52	-0.28241	-2.42299	0.0000	0.0109	Pfn1vscontrol_dnPfn1	msigdb_canonical
KEGG_NEUROACTIVE_LIGAND_RECEPTOR_INTERACTION	59	-0.2695	-2.39828	0.0020	0.0110	Pfn1vscontrol_dnPfn1	msigdb_canonical
KEGG_VASCULAR_SMOOTH_MUSCLE_CONTRACTION	42	-0.32012	-2.42991	0.0000	0.0114	Pfn1vscontrol_dnPfn1	msigdb_canonical
REACTOME_LIPID_DIGESTION_MOBILIZATION_AND_TRANSPORT	14	-0.51032	-2.35116	0.0022	0.0148	Pfn1vscontrol_dnPfn1	msigdb_canonical
KEGG_TASTE_TRANSDUCTION	14	-0.51154	-2.2884	0.0020	0.0213	Pfn1vscontrol_dnPfn1	msigdb_canonical
KEGG_FC_EPSILON_RI_SIGNALING_PATHWAY	37	-0.32223	-2.29055	0.0000	0.0222	Pfn1vscontrol_dnPfn1	msigdb_canonical
KEGG_ERBB_SIGNALING_PATHWAY	39	-0.30726	-2.23896	0.0020	0.0294	Pfn1vscontrol_dnPfn1	msigdb_canonical
KEGG_GNRH_SIGNALING_PATHWAY	48	-0.26889	-2.20176	0.0020	0.0348	Pfn1vscontrol_dnPfn1	msigdb_canonical
REACTOME_GASTRIN_CREB_SIGNALLING_PATHWAY_VIA_PKC_AND_MAPK	49	-0.26869	-2.15789	0.0019	0.0430	Pfn1vscontrol_dnPfn1	msigdb_canonical
REACTOME_PHASE1_FUNCTIONALIZATION_OF_COMPOUNDS	16	-0.42013	-2.05112	0.0039	0.0713	Pfn1vscontrol_dnPfn1	msigdb_canonical
KEGG_OOCYTE_MEIOSIS	48	-0.25248	-2.0562	0.0000	0.0718	Pfn1vscontrol_dnPfn1	msigdb_canonical
REACTOME_PHOSPHOLIPASE_C_MEDIATED_CASCADE	21	-0.37522	-2.06639	0.0082	0.0742	Pfn1vscontrol_dnPfn1	msigdb_canonical
KEGG_DRUG_METABOLISM_CYTOCHROME_P450	11	-0.50753	-2.05757	0.0057	0.0746	Pfn1vscontrol_dnPfn1	msigdb_canonical
KEGG_MELANOGENESIS	37	-0.28274	-2.03455	0.0040	0.0758	Pfn1vscontrol_dnPfn1	msigdb_canonical
REACTOME_DEPOSITION_OF_NEW_CENPA_CONTAINING_NUCLEOSOMES_AT_THE_CENTROMERE	10	-0.52334	-2.01811	0.0022	0.0795	Pfn1vscontrol_dnPfn1	msigdb_canonical
REACTOME_G_ALPHA_I_SIGNALLING_EVENTS	46	-0.24535	-1.97584	0.0063	0.0972	Pfn1vscontrol_dnPfn1	msigdb_canonical
IL21_UP.V1_UP	45	-0.3534	-2.79575	0.0000	0.0000	Pfn1vscontrol_dnPfn1	msigdb_oncogenicsignatures
PRC1_BMI_UP.V1_DN	44	-0.32838	-2.57182	0.0000	0.0034	Pfn1vscontrol_dnPfn1	msigdb_oncogenicsignatures
PTEN_DN.V1_UP	43	-0.34206	-2.58702	0.0000	0.0051	Pfn1vscontrol_dnPfn1	msigdb_oncogenicsignatures
KRAS.600_UP.V1_DN	58	-0.27615	-2.42794	0.0000	0.0082	Pfn1vscontrol_dnPfn1	msigdb_oncogenicsignatures
ATF2_S_UP.V1_UP	50	-0.28847	-2.36688	0.0000	0.0085	Pfn1vscontrol_dnPfn1	msigdb_oncogenicsignatures
IL2_UP.V1_UP	54	-0.27151	-2.37165	0.0020	0.0100	Pfn1vscontrol_dnPfn1	msigdb_oncogenicsignatures
PRC1_BMI_UP.V1_UP	45	-0.29213	-2.30192	0.0020	0.0102	Pfn1vscontrol_dnPfn1	msigdb_oncogenicsignatures
ATF2_UP.V1_UP	59	-0.25959	-2.3266	0.0000	0.0105	Pfn1vscontrol_dnPfn1	msigdb_oncogenicsignatures
KRAS.600.LUNG.BREAST_UP.V1_DN	65	-0.23879	-2.2561	0.0000	0.0127	Pfn1vscontrol_dnPfn1	msigdb_oncogenicsignatures
BMI1_DN_MEL18_DN.V1_UP	46	-0.27356	-2.22161	0.0040	0.0151	Pfn1vscontrol_dnPfn1	msigdb_oncogenicsignatures
KRAS.DF.V1_DN	58	-0.24618	-2.17589	0.0020	0.0170	Pfn1vscontrol_dnPfn1	msigdb_oncogenicsignatures
KRAS.AMP.LUNG_UP.V1_UP	26	-0.36786	-2.18785	0.0020	0.0172	Pfn1vscontrol_dnPfn1	msigdb_oncogenicsignatures
BMI1_DN.V1_UP	42	-0.27441	-2.13396	0.0020	0.0206	Pfn1vscontrol_dnPfn1	msigdb_oncogenicsignatures
NOTCH_DN.V1_DN	42	-0.27481	-2.11035	0.0020	0.0214	Pfn1vscontrol_dnPfn1	msigdb_oncogenicsignatures
PRC2_SUZ12_UP.V1_DN	37	-0.29242	-2.09766	0.0019	0.0218	Pfn1vscontrol_dnPfn1	msigdb_oncogenicsignatures
RELA_DN.V1_DN	26	-0.32574	-2.00552	0.0061	0.0307	Pfn1vscontrol_dnPfn1	msigdb_oncogenicsignatures
PKCA_DN.V1_UP	27	-0.3223	-1.99393	0.0000	0.0310	Pfn1vscontrol_dnPfn1	msigdb_oncogenicsignatures
MEL18_DN.V1_UP	46	-0.2492	-2.00911	0.0039	0.0316	Pfn1vscontrol_dnPfn1	msigdb_oncogenicsignatures
BRCA1_DN.V1_UP	30	-0.30925	-1.98185	0.0083	0.0322	Pfn1vscontrol_dnPfn1	msigdb_oncogenicsignatures
ATM_DN.V1_DN	37	-0.27992	-2.01989	0.0059	0.0327	Pfn1vscontrol_dnPfn1	msigdb_oncogenicsignatures
PIGF_UP.V1_DN	54	-0.23022	-2.0096	0.0039	0.0334	Pfn1vscontrol_dnPfn1	msigdb_oncogenicsignatures
AKT_UP_MTOR_DN.V1_UP	48	-0.2351	-1.9375	0.0123	0.0402	Pfn1vscontrol_dnPfn1	msigdb_oncogenicsignatures
CTIP_DN.V1_DN	37	-0.2682	-1.92195	0.0077	0.0423	Pfn1vscontrol_dnPfn1	msigdb_oncogenicsignatures
P53_DN.V2_UP	41	-0.25173	-1.89113	0.0062	0.0465	Pfn1vscontrol_dnPfn1	msigdb_oncogenicsignatures
CYCLIN_D1_KE_.V1_DN	68	-0.2004	-1.89168	0.0061	0.0482	Pfn1vscontrol_dnPfn1	msigdb_oncogenicsignatures
P53_DN.V2_DN	42	-0.24121	-1.85556	0.0021	0.0551	Pfn1vscontrol_dnPfn1	msigdb_oncogenicsignatures
IL15_UP.V1_UP	54	-0.21141	-1.84587	0.0080	0.0553	Pfn1vscontrol_dnPfn1	msigdb_oncogenicsignatures
BRCA1_DN.V1_DN	36	-0.25538	-1.82006	0.0204	0.0616	Pfn1vscontrol_dnPfn1	msigdb_oncogenicsignatures
PRC2_SUZ12_UP.V1_UP	57	-0.20621	-1.80149	0.0174	0.0657	Pfn1vscontrol_dnPfn1	msigdb_oncogenicsignatures
JAK2_DN.V1_UP	39	-0.23853	-1.78752	0.0203	0.0685	Pfn1vscontrol_dnPfn1	msigdb_oncogenicsignatures
SNF5_DN.V1_DN	45	-0.22179	-1.77437	0.0261	0.0715	Pfn1vscontrol_dnPfn1	msigdb_oncogenicsignatures
JNK_DN.V1_UP	43	-0.22491	-1.72471	0.0232	0.0886	Pfn1vscontrol_dnPfn1	msigdb_oncogenicsignatures
KRAS.AMP.LUNG_UP.V1_DN	29	-0.27087	-1.71856	0.0346	0.0887	Pfn1vscontrol_dnPfn1	msigdb_oncogenicsignatures
ATM_DN.V1_UP	51	-0.2007	-1.70484	0.0224	0.0928	Pfn1vscontrol_dnPfn1	msigdb_oncogenicsignatures
NOTCH_DN.V1_UP	32	-0.25893	-1.69808	0.0318	0.0932	Pfn1vscontrol_dnPfn1	msigdb_oncogenicsignatures
KRAS.300_UP.V1_DN	28	-0.27558	-1.68928	0.0276	0.0949	Pfn1vscontrol_dnPfn1	msigdb_oncogenicsignatures
KEGG_TYPE_I_DIABETES_MELLITUS	20	0.469099	2.57802	0.0000	0.0133	Pfn1vscontrol_upPfn1	msigdb_canonical
REACTOME_ASPARAGINE_N_LINKED_GLYCOSYLATION	27	0.424601	2.619969	0.0000	0.0194	Pfn1vscontrol_upPfn1	msigdb_canonical
KEGG_PROTEASOME	22	0.420411	2.349091	0.0040	0.0231	Pfn1vscontrol_upPfn1	msigdb_canonical
REACTOME_METABOLISM_OF_PROTEINS	116	0.197891	2.442641	0.0000	0.0253	Pfn1vscontrol_upPfn1	msigdb_canonical
REACTOME_ASSEMBLY_OF_THE_PRE_REPLICATIVE_COMPLEX	25	0.385951	2.355027	0.0000	0.0267	Pfn1vscontrol_upPfn1	msigdb_canonical
REACTOME_POST_TRANSLATIONAL_PROTEIN_MODIFICATION	54	0.277201	2.381779	0.0000	0.0277	Pfn1vscontrol_upPfn1	msigdb_canonical
REACTOME_VIF_MEDIATED_DEGRADATION_OF_APOBEC3G	25	0.385951	2.267918	0.0000	0.0320	Pfn1vscontrol_upPfn1	msigdb_canonical
REACTOME_P53_INDEPENDENT_G1_S_DNA_DAMAGE_CHECKPOINT	24	0.382517	2.248793	0.0039	0.0332	Pfn1vscontrol_upPfn1	msigdb_canonical
KEGG_ALLOGRAFT_REJECTION	16	0.461192	2.236285	0.0020	0.0332	Pfn1vscontrol_upPfn1	msigdb_canonical
REACTOME_METABOLISM_OF_RNA	81	0.21768	2.271514	0.0020	0.0358	Pfn1vscontrol_upPfn1	msigdb_canonical
REACTOME_ER_PHAGOSOME_PATHWAY	27	0.358192	2.20953	0.0000	0.0369	Pfn1vscontrol_upPfn1	msigdb_canonical
REACTOME_ANTIGEN_PROCESSING_CROSS_PRESENTATION	33	0.324745	2.184109	0.0020	0.0393	Pfn1vscontrol_upPfn1	msigdb_canonical
REACTOME_AUTODEGRADATION_OF_THE_E3_UBIQUITIN_LIGASE_COP1	23	0.378794	2.140874	0.0000	0.0419	Pfn1vscontrol_upPfn1	msigdb_canonical

KEGG_GRAFT_VERSUS_HOST_DISEASE	19	0.400298	2.15112	0.0037	0.0419	Pfn1vscontrol_upPfn1	msigdb_canonical
REACTOME_HIV_INFECTION	90	0.19085	2.152652	0.0000	0.0447	Pfn1vscontrol_upPfn1	msigdb_canonical
REACTOME_REGULATION_OF_ORNITHINE_DECARBOXYLASE_ODC	26	0.350451	2.123361	0.0039	0.0447	Pfn1vscontrol_upPfn1	msigdb_canonical
REACTOME_M_G1_TRANSITION	29	0.32799	2.09681	0.0019	0.0469	Pfn1vscontrol_upPfn1	msigdb_canonical
REACTOME_ACTIVATION_OF_NF_KAPPAB_IN_B_CELLS	29	0.32799	2.089923	0.0019	0.0469	Pfn1vscontrol_upPfn1	msigdb_canonical
REACTOME_MHC_CLASS_II_ANTIGEN_PRESENTATION	32	0.305884	2.082609	0.0019	0.0470	Pfn1vscontrol_upPfn1	msigdb_canonical
REACTOME_CDT1_ASSOCIATION_WITH_THE_CDC6_ORC_ORIGIN_COMPLEX	22	0.374741	2.07132	0.0020	0.0480	Pfn1vscontrol_upPfn1	msigdb_canonical
REACTOME_METABOLISM_OF_MRNA	63	0.215044	2.028193	0.0101	0.0480	Pfn1vscontrol_upPfn1	msigdb_canonical
REACTOME_MITOTIC_M_M_G1_PHASES	56	0.228281	2.01664	0.0040	0.0481	Pfn1vscontrol_upPfn1	msigdb_canonical
REACTOME_TRANSLATION	36	0.288114	2.011037	0.0019	0.0482	Pfn1vscontrol_upPfn1	msigdb_canonical
REACTOME_DOWNSTREAM_SIGNALING_EVENTS_OF_B_CELL_RECEPTOR_BCR	38	0.273351	2.022125	0.0020	0.0483	Pfn1vscontrol_upPfn1	msigdb_canonical
REACTOME_SCF_BETA_TRCP_MEDIATED_DEGRADATION_OF_EMI1	22	0.374741	2.034357	0.0059	0.0484	Pfn1vscontrol_upPfn1	msigdb_canonical
KEGG_INTESTINAL_IMMUNE_NETWORK_FOR_IGA_PRODUCTION	19	0.395431	2.046611	0.0021	0.0487	Pfn1vscontrol_upPfn1	msigdb_canonical
REACTOME_SRP_DEPENDENT_COTRANSLATIONAL_PROTEIN_TARGETING_TO_MEMBRANE	23	0.352718	2.051531	0.0020	0.0494	Pfn1vscontrol_upPfn1	msigdb_canonical
REACTOME_APC_C_CDH1_MEDIATED_DEGRADATION_OF_CDC20_AND_OTHER_APC_C_CDH1_TARGETED_PROTEINS_IN_LATE_MITOSIS_EARLY_G1	31	0.298378	1.995977	0.0075	0.0494	Pfn1vscontrol_upPfn1	msigdb_canonical
REACTOME_DESTABILIZATION_OF_MRNA_BY_AUF1_HNRNP_D0	22	0.374741	2.097072	0.0000	0.0495	Pfn1vscontrol_upPfn1	msigdb_canonical
REACTOME_CROSS_PRESENTATION_OF_SOLUBLE_EXOGENOUS_ANTIGENS_ENDOSOMES	24	0.340636	1.999427	0.0020	0.0498	Pfn1vscontrol_upPfn1	msigdb_canonical
KEGG_AUTOIMMUNE_THYROID_DISEASE	15	0.444373	2.057086	0.0000	0.0498	Pfn1vscontrol_upPfn1	msigdb_canonical
KEGG_LEISHMANIA_INFECTION	32	0.304916	2.035649	0.0042	0.0499	Pfn1vscontrol_upPfn1	msigdb_canonical
KEGG_N_GLYCAN_BIOSYNTHESIS	14	0.428725	1.986591	0.0060	0.0506	Pfn1vscontrol_upPfn1	msigdb_canonical
BIOCARTA_PROTEASOME_PATHWAY	13	0.441183	1.931478	0.0057	0.0662	Pfn1vscontrol_upPfn1	msigdb_canonical
REACTOME_HOST_INTERACTIONS_OF_HIV_FACTORS	56	0.22065	1.933946	0.0085	0.0675	Pfn1vscontrol_upPfn1	msigdb_canonical
KEGG_ANTIGEN_PROCESSING_AND_PRESENTATION	30	0.289247	1.904123	0.0080	0.0761	Pfn1vscontrol_upPfn1	msigdb_canonical
REACTOME_REGULATION_OF_MRNA_STABILITY_BY_PROTEINS_THAT_BIND_AU_RICH_ELEMENTS	34	0.271074	1.886372	0.0039	0.0814	Pfn1vscontrol_upPfn1	msigdb_canonical
REACTOME_APC_C_CDC20_MEDIATED_DEGRADATION_OF_MITOTIC_PROTEINS	28	0.291733	1.867156	0.0101	0.0876	Pfn1vscontrol_upPfn1	msigdb_canonical
KEGG_CELL_ADHESION_MOLECULES_CAMS	43	0.241089	1.857954	0.0142	0.0899	Pfn1vscontrol_upPfn1	msigdb_canonical
KEGG_AMINOACYL_TRNA_BIOSYNTHESIS	11	0.466812	1.845992	0.0177	0.0916	Pfn1vscontrol_upPfn1	msigdb_canonical
REACTOME_CDK_MEDIATED_PHOSPHORYLATION_AND_REMOVAL_OF_CDC6	23	0.335101	1.829268	0.0163	0.0917	Pfn1vscontrol_upPfn1	msigdb_canonical
KEGG_PORPHYRIN_AND_CHLOROPHYLL_METABOLISM	13	0.420827	1.831029	0.0177	0.0930	Pfn1vscontrol_upPfn1	msigdb_canonical
REACTOME_TRNA_AMINOACYLATION	11	0.466812	1.835019	0.0136	0.0932	Pfn1vscontrol_upPfn1	msigdb_canonical
KEGG_ASTHMA	12	0.442588	1.846919	0.0097	0.0933	Pfn1vscontrol_upPfn1	msigdb_canonical
PID_EPHB_FWD_PATHWAY	25	0.309474	1.837637	0.0076	0.0938	Pfn1vscontrol_upPfn1	msigdb_canonical
PID_IL27_PATHWAY	12	0.43102	1.809487	0.0196	0.0993	Pfn1vscontrol_upPfn1	msigdb_canonical
JAK2_DN.V1_DN	47	0.328719	2.706933	0.0000	0.0027	Pfn1vscontrol_upPfn1	msigdb_oncogenicsignatures
PTEN_DN.V1_DN	45	0.236893	1.928231	0.0042	0.0960	Pfn1vscontrol_upPfn1	msigdb_oncogenicsignatures
CAMP_UP.V1_UP	63	0.222129	2.081649	0.0019	0.1008	Pfn1vscontrol_upPfn1	msigdb_oncogenicsignatures
GCNP_SHH_UP_EARLY.V1_UP	52	0.229473	1.956273	0.0040	0.1038	Pfn1vscontrol_upPfn1	msigdb_oncogenicsignatures
