# Supplementary material for: Profilin-1 deficiency leads to SMAD3 upregulation and impaired 3D outgrowth of breast cancer cells
Source: Br J Cancer. 2018 Oct 15;119(9):1106–17. doi: 10.1038/s41416-018-0284-6 (PMC6219497; doi:10.1038/s41416-018-0284-6)
Supplement: Supplementary file 13 — Supplementary table S2 [file 41416_2018_284_MOESM13_ESM.pdf]

Supplementary Table 2

Upstream Regulator	Exp Log Ratio	Molecule Type	Predicted Activation State	Activation z-score	Bias Term	Bias-corrected z-score	p-value of overlap	Target molecules in dataset
rosiglitazone		chemical drug	Inhibited	-2.611	-0.019	-2.55	1.31E-02	CEBPA,CLEC7A,DIO2,EBI3,FABP3,MLYCD,MR1,PLIN5,TERT,UCP3
fenofibrate		chemical drug	Inhibited	-2.408	-0.015	-2.371	1.47E-02	APOA1,APOB,FADS2,MLYCD,TERT,TIMM17A,UCP3
miR-4308 (and other miRNAs w/seed CCCUGGA)		mature microrna	Inhibited	-2.216	0.053	-2.334	1.53E-01	ARHGAP30,C11orf63,SYN2,SYT12,VPS13C
miR-1913 (and other miRNAs w/seed CUGCCCC)		mature microrna	Inhibited	-2.178	0.053	-2.327	2.30E-01	ATP8B2,BCL11A,DPYSL3,KMT2D,PGP,SYT12,TMLHE,ZDHHC8
pirinixic acid		chemical toxicant	Inhibited	-2.285	-0.033	-2.193	1.73E-02	APOA1,FADS2,FCER1G,ITGB5,MAP3K8,MLYCD,PLIN5,TIMM17A,TUFM,UCP3
IL10		cytokine	Inhibited	-2.178	-0.006	-2.161	3.21E-02	CCND2,CDK6,CLEC7A,FCER1G,MAP3K8,MUC5AC,NFKBID,TERT
cyclic AMP		chemical - endogenous mammalian	Inhibited	-2.216	-0.034	-2.14	1.35E-01	CCND2,CEBPA,INHBA,TF,TYRP1
miR-1260a (and other miRNAs w/seed UCCCACC)		mature microrna	Inhibited	-2	0.053	-2.107	4.13E-01	ABHD13,FAM120C,TIMM17A,ZNF780B
miR-3194-3p (miRNAs w/seed GCUCUGC)		mature microrna		-1.982	0.053	-2.089	1.39E-01	ASPN,ATP8B2,MMP16,PGP
miR-875-5p (and other miRNAs w/seed AUACCUC)		mature microrna		-1.98	0.053	-2.086	7.17E-02	BCL11A,REV1,TLDC1,WNK3
miR-1258-5p (and other miRNAs w/seed GCUGAGC)		mature microrna		-1.981	0.053	-2.086	8.78E-02	C9orf3,FOXK1,SIRT5,WNK3
RICTOR		other	Inhibited	-2	0.039	-2.077	2.97E-01	ATP6V1C2,ATP6V1D,PPA2,PSMA8
SPDEF		transcription regulator	Inhibited	-2	0.035	-2.069	5.65E-03	COL6A1,COL6A3,MUC5AC,UCP3
miR-3176 (and other miRNAs w/seed CUGGCCU)		mature microrna		-1.964	0.053	-2.069	2.24E-01	ATF7IP2,GIPC3,ITGB5,ZNF641
miR-3127-3p (and other miRNAs w/seed CCCCUUC)		mature microrna		-1.951	0.053	-2.055	2.33E-01	C2orf88,DDX6,OKI,TMLHE
miR-335-5p (and other miRNAs w/seed CAAGAGC)		mature microrna		-1.912	0.053	-2.053	3.36E-02	ITGB8,PCNX1,SMARCA2,SPTSSA,SYN2,TRIM59,ZDHHC8
KDM5B		transcription regulator		-1.987	0.026	-2.038	5.37E-02	ESR1,NOL3,SMARCA2,SPTSSA
metformin		chemical drug		-1.981	0.018	-2.016	6.11E-02	APOB,CCND2,SIRT5,TERT
BCL6		transcription regulator		-1.964	0.016	-1.996	6.63E-02	CCND2,EBI3,Igk,SOX6
NKX2-3		transcription regulator		-1.987	0	-1.986	1.87E-01	MTURN,SRPX,TNFSF18,UCP3
SPI1		transcription regulator		-1.969	-0.021	-1.927	2.17E-02	BCL11A,CCND2,CEBPA,FCER1G,Igk,MBP
EDN1		cytokine	Inhibited	-2	-0.042	-1.915	1.55E-01	INHBA,MBP,PLCB4,TYRP1
PTEN		phosphatase		-1.897	0.003	-1.904	4.88E-01	CDK6,CEBPA,ESR1,HLA-DMA,MBP
SYVN1		transporter	Inhibited	-2	-0.05	-1.899	5.37E-02	CDA,DIO2,GPRC5A,STOM
L-dopa		chemical - endogenous mammalian		-1.912	-0.006	-1.897	1.00E+00	ADCK4,CASZ1,CSMD3,MBP,NOL3,SYN2,SYT12
EGR2		transcription regulator		-1.982	-0.047	-1.889	7.31E-02	DIO2,MBP,MR1,UCP3
CREB1		transcription regulator		-1.929	-0.017	-1.876	2.79E-02	CDA,DIO2,EBI3,INHBA,MUC5AC,NFKBID,NRSN1,SRD5A1,STC1,SYN2,TF
miR-128-3p (and other miRNAs w/seed CACAGUG)		mature microrna		-1.631	0.053	-1.852	3.29E-02	ALG9,BHLHE41,CPEB3,DDX6,FAM120C,FAXC,FOXN4,GAREM1,KIAA1109,MTURN,PCNX1,PDE10A,PLAGL1,OKI,SEMA6D,SMARCA2,UST,WNK3
miR-383-5p (miRNAs w/seed GAUCAGA)		mature microrna		-1.673	0.053	-1.801	2.76E-02	CCND2,CLEC3A,MALL,MESDC2,PCNX1,OKI
miR-3156-3p (miRNAs w/seed UCCCACU)		mature microrna		-1.633	0.053	-1.764	9.40E-03	ANKRD44,DDX6,HTRA4,RFX3,TUFM,ZNF780B
miR-4638-3p (and other miRNAs w/seed CUGGACA)		mature microrna		-1.558	0.053	-1.676	1.24E-02	INHBA,PSMA8,OKI,SYT12,VPS13C
PPARA		ligand-dependent nuclear receptor		-1.716	-0.016	-1.67	1.39E-02	APOA1,CEBPA,DIO2,FABP3,FADS2,MLYCD,PLIN5,SIRT5,TERT,UCP3
miR-1296-5p (miRNAs w/seed UAGGGCC)		mature microrna		-1.547	0.053	-1.664	1.02E-02	ALG9,C2orf88,NRCAM,SYN2,TRIM59
miR-5095 (and other miRNAs w/seed UACAGGC)		mature microrna		-1.436	0.053	-1.601	8.61E-03	C11orf63,C11orf70,CD84,DZANK1,MESDC2,MYO6,NUMBL,POLH,PPA2,TLDC1
miR-320b (and other miRNAs w/seed AAAGCUG)		mature microrna		-1.36	0.053	-1.6	1.18E-04	ABHD13,ALG9,ATF7IP2,ATL3,B4GALT6,BHLHE41,C8orf88,CCND2,CDK6,CPEB3,DPYSL3,FAM120C,GOLGA1,MAGI1,MBNL3,MLLT3,MMP16,PDE10A,PTBP3,SPTSSA,UBE2D3
miR-3194-5p (miRNAs w/seed GCCAGCC)		mature microrna		-1.394	0.053	-1.561	4.26E-02	ATP8B2,CASP14,CSMD3,DZANK1,FAM120C,KMT2D,NUMBL,RFFL,TLDC1,TMPRSS13
decitabine		chemical drug		-1.657	-0.041	-1.539	1.27E-02	CASZ1,CCND2,COL6A3,DAPK1,EGFL6,ESR1,GPR85,HELLS,KIR2DL2,SMARCA2,TERT
Vegf		group		1.419	-0.039	1.539	3.51E-02	ENPP2,HELLS,IL11,INHBA,ITGB8,MAGI1,MALL,MAP3K8,NRCAM,STC1
miR-3175 (miRNAs w/seed GGGGAGA)		mature microrna		1.784	0.053	1.646	2.89E-01	ACTL10,DIO2,FADS2,GPRC5A,PDE6G,PRKAR1B,WNK3
raloxifene		chemical drug		1.698	0	1.698	9.40E-03	CD24,ENPP2,ESR1,GPRC5A,STC1,TERT
miR-3173-5p (and other miRNAs w/seed GCCCUGC)		mature microrna		1.895	0.053	1.747	7.54E-02	CASZ1,CD84,CEP126,FAM107A,IBA57,MBNL3,MR1,PXDC1
miR-103-3p (and other miRNAs w/seed GCAGCAU)		mature microrna		1.957	0.053	1.749	5.20E-03	ACTR2,ANKRD44,BCL11A,CDK6,CHD6,CPEB3,DPYSL3,ESR1,HUWE1,KMT2D,MBNL3,SIPA1L2,SOX6,TRIM59,UBE2O1,WNK3
miR-361-3p (miRNAs w/seed CCCCCAG)		mature microrna		1.912	0.053	1.772	3.32E-01	CEBPA,CSNK1G1,EMX1,GSE1,IL11,KIAA1644,SYN2
miR-149-5p (miRNAs w/seed CUGGCUC)		mature microrna		1.961	0.053	1.793	9.51E-02	CDK6,CSNK1G1,FAM120C,FOXN4,GAREM1,IL11,KMT2D,MTHFR,TSPAN33,ZNF335
miR-6967-5p (and other miRNAs w/seed AGGGAGG)		mature microrna		1.947	0.053	1.809	3.52E-02	CASP14,HIF3A,MBP,PDE6G,RFFL,UBE2O1,UCP3
miR-4769-3p (and other miRNAs w/seed CUGCCAU)		mature microrna		1.934	0.053	1.831	1.84E-02	CSNK1G1,HOMEZ,MBNL3,RADIL
miR-1972 (miRNAs w/seed CAGGCCA)		mature microrna		1.96	0.053	1.855	2.81E-01	ALX4,DOPEY1,MR1,MTHFR
CTNNB1		transcription regulator		1.761	-0.043	1.863	1.09E-01	CCND2,CEBPA,COL27A1,DIO2,ENPP2,FCER1G,FGD2,GRIK1,MMP16,NRCAM,TERT
fulvestrant		chemical drug		1.969	0.012	1.945	2.85E-01	APOA1,ESR1,RNF126,STC1
miR-342-5p (and other miRNAs w/seed GGGGUGC)		mature microrna	Activated	2.138	0.053	1.987	5.63E-02	FAM107A,FOXN4,GOLGA1,GPRC5A,PDE6G,SYNPO2,ZDHHC8,ZNF395
INHBA	-3.398	growth factor		1.994	-0.028	2.051	1.23E-01	CCND2,CD24,ESR1,INHBA
SMAD3		transcription regulator		1.973	-0.028	2.059	4.01E-06	APOA1,APOB,ASPN,CCND2,COL6A1,COL6A3,DAPK1,DOCK4,ESR1,ITGB5,TERT,TF
